# Genome-wide identification and stress response analysis of cyclophilin gene family in apple (*Malus × domestica*)

**DOI:** 10.1186/s12864-022-08976-w

**Published:** 2022-12-06

**Authors:** Zhi-Wen Qiao, Da-Ru Wang, Xun Wang, Chun-Xiang You, Xiao-Fei Wang

**Affiliations:** State Key Laboratory of Crop Biology, College of Horticulture Science and Engineering, Shandong Agricultural University, Tai’an, 271018 Shandong China

**Keywords:** *Malus × domestica*, Cyclophilin, CYP, Abiotic stress

## Abstract

**Background:**

Cyclophilin (CYP) belongs to the immunophilin family and has peptidyl-prolyl *cis*-*trans* isomerase (PPIase) activity, which catalyzes the *cis*-*trans* isomerization process of proline residues. CYPs widely exist in eukaryotes and prokaryotes, and contain a conserved cyclophilin-like domain (CLD). Plant cyclophilins are widely involved in a range of biological processes including stress response, metabolic regulation, and growth and development.

**Result:**

In this study, 30 cyclophilin genes on 15 chromosomes were identified from the ‘Golden Delicious’ apple (*M. domestica*) genome. Phylogenetic analysis showed that the cyclophilin family genes can be divided into three clades in *Malus*. Collinear analysis showed that ten gene pairs were the result of segmental duplication. Analysis of gene and protein structure further supported the phylogenetic tree and collinearity analysis. The expression of *MdCYPs* in different organs was higher in leaves, flowers, and fruits. Ten and eight CYPs responded to drought and salt stress, respectively. MdCYP16, a nuclear-localized MD CYP, was screened from the intersection of the two expression profiling datasets and was highly sensitive to drought and salt stress. GUS staining of transgenic *Arabidopsis* indicated that *MdCYP16* may be involved in the regulation of abiotic stress.

**Conclusion:**

This study systematically analyzed members of the apple cyclophilin family and confirmed the involvement of *MdCYP16* as a nuclear-localized MD cyclophilin that acts in response to salt and drought stress in apple. Our work identifies members of the apple cyclophilin gene family, and provides an important theoretical basis for in-depth study of cyclophilin function. Additionally, the analysis provides candidate genes that may be involved in stress response in apple.

**Supplementary Information:**

The online version contains supplementary material available at 10.1186/s12864-022-08976-w.

## Background

Cyclophilin (CYP) proteins widely exist in bacteria, fungi, plants, and animals, and are part of an immunophilin superfamily with FK506-binding proteins FKBPs and Parvulins [1–3]. CYPs possess peptidyl-prolyl *cis-trans* isomerase (PPIase) activity, catalyzing the rotation of Xaa-Proline peptide bonds from a *cis* to *trans* conformation, often a rate-limiting step in protein folding [4, 5]. As molecular chaperones, CYPs act in a wide range of biological processes, including protein transport, transcriptional regulation, signal transduction, mRNA splicing, cell apoptosis, and stress responses to biological and abiotic stresses [6–8].

In 1984, the first CYP protein was identified and purified from bovine thymocytes as an immunosuppressant cyclosporin A (CsA) -specific receptor protein [9]. Plant CYPs were first identified in tomato, maize, and *Brassica napus* in 1990 [10]. Since then, plant CYPs have been identified and studied in many species, including 35 *AtCYPs* in *Arabidopsis* [11, 12], 28 *OsCYPs* in rice [12], 62 *GmCYPs* in soybean [13], 39 *ZmCYPs* in maize [14], 94 *BnCYPs* in oilseed rape [15], 79 *GhCYPs* in cotton [16], and 33 Ms*CYPs* in alfalfa [17]. Members of the plant CYP family are highly conserved due to the presence of a cyclophilin-like domain (CLD), and proteins can be subdivided into single-domain (SD) and multi-domain (MD) groups. Some SD CYPs have localization sequences at their N-termini and some possess predicted transmembrane domains (TMD) at the N- and C-termini. In addition to a CLD domain, different MD CYPs contain a variety of other functional domains, such as tetratricopeptide repeat (TPR), U-box, WD-40 repeat, RNA recognition motif (RRM), and zinc finger domain [11].

In higher plants, CYPs are involved in regulating a range of plant physiological processes. In development of organs and tissue, loss-of-function mutations of *CYP40* reduced the number of juvenile leaves [18], caused the precocious expression of adult vegetative traits, increased carpel number, and produced abnormal spacing of flowers in inflorescences [19]. CYP40 indirectly increases the activity of miR156 by increasing the activity of ARGONAUTE1 (AGO1), thus indirectly regulating the gene inhibition of SPL family members mediated by miR156. In addition, CYP40 interacts with cytoplasmic Hsp90 proteins but not with Hsp90 proteins localized to chloroplasts, mitochondria, or the endoplasmic reticulum [20]. A WD40 domain cyclophilin CYP71 interacts with histone H3 and functions in gene repression and organogenesis in *Arabidopsis* [21]. CYP71 also interacts with CAF-1 and LHP1 and functions in multiple chromatin remodeling processes [22]. CYP20-2 modulates the conformation of BRASSINAZOLE-RESISTANT1 (BZR1), which binds the promoter of FLOWERING LOCUS D (FLD) to regulate flowering in *Arabidopsis* [23]. LATERAL ROOTLESS2 is a cyclophilin protein that regulates lateral root initiation and auxin signaling pathway in rice [24]. OsCYP20-2 interacts with OsSYF2, a pre-mRNA splicing factor, and controls grain length and panicle architecture by regulating the alternative splicing of pre-mRNA involved in cell elongation and sugar metabolism [25]. These proteins can also regulate transcription. For example, AtCYP59 interacts with the C-terminal domain (CTD) of the largest subunit of RNA polymerase II, connecting transcription and pre-mRNA processing [26]. In the process of photosynthesis, several CYPs have been localized to chloroplast thylakoids. Spinach (*Spinacia oleracca*) TLP40 affects the dephosphorylation of key proteins of photosystem II (PSII) and may be part of the transmembrane signal transduction chain [27]. TLP20 is the major PPIase and protein folding catalyst in the thylakoid lumen of chloroplasts [28]. AtCYP20-2, the homolog of TLP20 in *Arabidopsis*, specifically associates with membrane regions enriched in PSII supercomplexes [29]. Other studies have found that AtCYP38 is necessary for the assembly and stabilization of PSII [30]. AtCYP20-3 links photosynthetic electron transport and redox regulation to the folding of serine acetyltransferase (SAT1), thereby enabling modulation of the cysteine-based thiol biosynthesis pathway in response to light and stress conditions [31]. In response to biotic stress, constitutive heterologous expression of *Arachis diogoi* gene *AdCyp* resulted in enhanced resistance to *Ralstonia solanacearum* and reduced susceptibility towards *Phytophthora parasitica* var. *nicotianae* in transgenic tobacco [32]. In response to abiotic stress, ectopic overexpression of the *ThCYP1* gene of *Thellungiella halophila* can enhance the salt tolerance capacity of tobacco [33]. Transgenic *Arabidopsis* expressing *Cajanus cajan* gene *CcCYP* exhibited high-level tolerance against drought, salinity, and extreme temperature stresses [34]. *OsCYP2* of rice was up-regulated under salt stress, with enhanced tolerance of transgenic rice seedlings to salt stress [35]. Overexpression of *GhCyp1* in transgenic cotton also conferred higher tolerance to salt stress [36]. Ectopic expression of rice *OsCYP20-2* in both tobacco and *Arabidopsis* confers enhanced tolerance to osmotic stress and extremely high light [37]. Transgenic rice overexpressing *OsCYP21-4* exhibited increased tolerance to salinity and hydrogen peroxide treatments, along with increased peroxidase activity [38]. Overexpression of *OsCYP18-2* in transgenic rice and *Arabidopsis* enhanced drought tolerance and altered expression and pre-mRNA splicing patterns of stress-related genes in *Arabidopsis* under drought conditions [39]. The loss-of function *OsCYP20-2* mutant showed sensitivity to chilling stress with accumulation of extra reactive oxygen species (ROS) [40]. The CYP AtROC1(S58F) confers *Arabidopsis* freezing tolerance by modulating both jasmonate signaling and antioxidant metabolism [41]. In additon, ROC3 (AtCYP19-1) positively regulates ABA-induced stomatal closure and the drought response by regulating ROS homeostasis and the expression of various stress-activated genes [42].

Plant CYPs have been extensively studied in model crops *Arabidopsis* and rice, but little is known about what these proteins do in horticultural crops. As a universally popular fruit, apple is widely produced all over the world. Selection and cultivation of high quality apple germplasm resources with strong resistance can increase production yield. Because CYPs play roles in resistance to stress, increased understanding of these proteins in apple could help facilitate the development of improved germplasm resources for increased production. Here, we identified 30 cyclophilin members from apple that are distributed on 15 chromosomes. We systematically explored the members of the MdCYP family from the perspectives of gene structure, protein structure, promoter elements, and phylogenetic and collinearity analysis. Next, the expression patterns of *MdCYPs* were analyzed in different organs and under abiotic stress. In addition, the function of *MdCYP16* was analyzed to preliminarily reveal a role in salt and drought stress. This study will provide new ideas for future studies on the role of apple CYP genes in stress tolerance, and provides more candidate genes for use in breeding for apple stress tolerance.

## Results

### Identification of cyclophilins in apple (*M. domestica)* genome

**Fig. 1:**
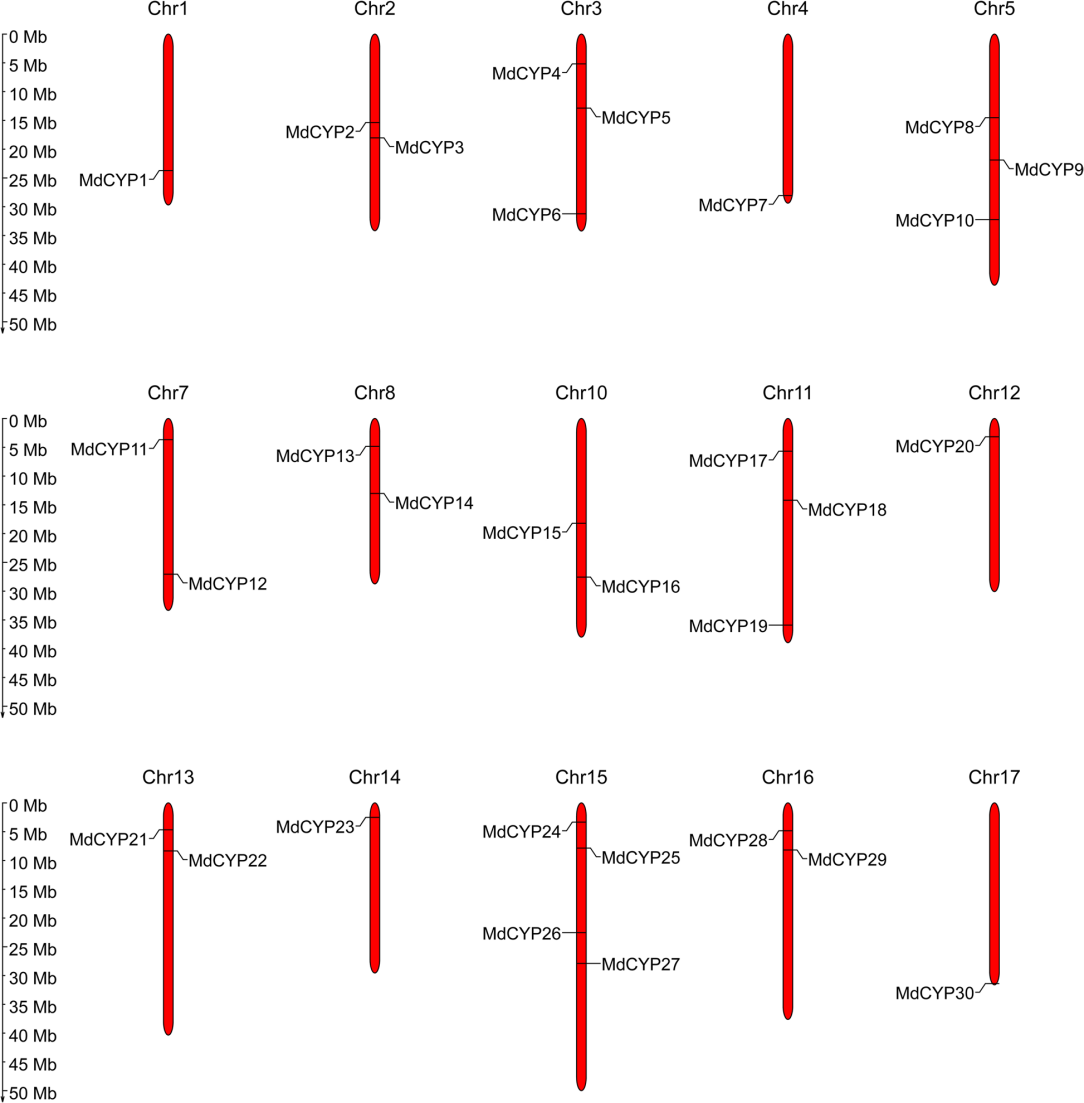
Chromosomal location of MdCYPs. Thirty MdCYP genes were distributed on 15 apple chromosomes. Chromosomal locations of MdCYPs are based on the physical positions (Mb) of genes from the GDDH13 Reference Genome annotation

The HMM (Hidden Markov Model) strategy was used to identify sequences matching the cyclophilin conserved CLD (PF00160) domain, followed by CLD confirmation using SMART. A total of 30 members were identified from the apple ‘Golden Delicious’ GDDH13 Reference Genome. These CYPs were distributed on 15 chromosomes (Fig. 1), and were named MdCYP1-30 according to the chromosomal positions of these genes (Table 1). The coding nucleic acid sequences length of MdCYP varied from 483 to 2556 base pairs, for predicted protein sequences that range from 160 to 851 amino acids. The maximum molecular weight (MW) was 95.7 kD, and the minimum MW was 17.20 kD. The theoretical isoelectric points ranged from 5.7 to 11.56, and were mostly alkaline. The instability index was greater than 40, indicating that these are all unstable proteins. The grand average hydropathicity values were negative, indicating that the MdCYPs are hydrophilic proteins. In addition, we visualized the predicted subcellular localization of MdCYP proteins and found 10 MdCYPs localized in the nucleus, nine MdCYPs localized in the cytoplasm, six MdCYPs localized in chloroplasts, three MdCYPs localized in vacuoles, one MdCYP in the cytoskeleton, and one extracellular MdCYP (Table 1).

**Table 1:** Information statistics of cyclophilin family members in apple (*M. domestica*)

Gene Name	Accession Number	Chromosomal Location	CDS(bp)	Protein(aa)	pI	MW(kD)	Ii	Ai	GRAVY	Subcellular localization prediction
MdCYP1	MD01G1152800	Chr1:26117518..26119871	678	225	6.53	24.48195	25.59	82.67	−0.136	vacuole
MdCYP2	MD02G1185800	Chr2:16878399..16882986	1788	595	7.97	65.17845	36.59	68.72	−0.53	nucleus
MdCYP3	MD02G1201300	Chr2:19895874..19897921	483	160	7.77	17.37477	15.84	78.69	−0.224	cytoplasmskeleton
MdCYP4	MD03G1070100	Chr3:5724914..5727412	615	204	9.15	21.85521	8.4	81.18	−0.121	extracellular
MdCYP5	MD03G1138000	Chr3:14095422..14104165	1911	636	6.23	71.58536	42.57	76.57	−0.448	nucleus
MdCYP6	MD03G1257000	Chr3:34422870..34433514	2100	699	11.1	78.74325	90.2	40.56	−1.476	nucleus
MdCYP7	MD04G1228500	Chr4:30894118..30895418	486	161	6.89	17.20053	10.43	61.12	−0.304	cytoplasm
MdCYP8	MD05G1075200	Chr5:16012977..16017802	1089	362	5.7	40.24664	21.81	73.29	−0.523	cytoplasm
MdCYP9	MD05G1119400	Chr5:24114220..24117701	675	224	7.11	24.88356	32.64	91.65	−0.096	chloroplast
MdCYP10	MD05G1222400	Chr5:35494362..35501420	1926	641	5.84	74.00101	50.29	56.61	−1.266	nucleus
MdCYP11	MD07G1046700	Chr7:3974977..3978052	576	191	8.15	20.58666	23.67	74.55	−0.129	chloroplast
MdCYP12	MD07G1221700	Chr7:29879243..29881526	681	226	8.59	24.48718	20.94	87.88	−0.108	vacuole
MdCYP13	MD08G1066000	Chr8:5279500..5281237	813	270	7.1	30.13709	32.03	73.59	−0.658	nucleus
MdCYP14	MD08G1145000	Chr8:14409053..14416828	2556	851	11.58	95.73674	122.02	42.35	−1.381	nucleus
MdCYP15	MD10G1123000	Chr10:20043401..20046958	675	224	6.71	24.81751	28.51	90.36	−0.083	cytoplasm
MdCYP16	MD10G1204000	Chr10:30303123..30316243	1887	628	5.96	72.36817	45.88	54.67	−1.278	nucleus
MdCYP17	MD11G1073900	Chr11:6253081..6255477	615	204	8.99	21.79312	9.48	84.56	−0.082	vacuole
MdCYP18	MD11G1160100	Chr11:15695667..15713322	1896	631	6.2	71.09686	42.47	76.88	−0.439	cytoplasm
MdCYP19	MD11G1277600	Chr11:39477241..39482269	2034	677	10.99	75.46333	78.99	38.15	−1.467	nucleus
MdCYP20	MD12G1030900	Chr12:3429752..3432621	495	164	8.43	18.1676	26.98	70.73	−0.463	cytoplasm
MdCYP21	MD13G1074200	Chr13:5239114..5242164	732	243	7.76	27.33819	46.24	73.42	−0.272	chloroplast
MdCYP22	MD13G1123100	Chr13:9128856..9131629	780	259	9.41	27.43002	36.22	74.83	−0.186	chloroplast
MdCYP23	MD14G1031400	Chr14:2806135..2809153	495	164	8.76	18.13363	23.57	72.5	−0.457	cytoplasm
MdCYP24	MD15G1055400	Chr15:3743782..3747204	1494	497	7.69	56.24827	41.69	70.8	−0.969	cytoplasm
MdCYP25	MD15G1120600	Chr15:8676138..8681402	1305	434	9.54	48.91945	64.26	53.89	−1.148	nucleus
MdCYP26	MD15G1280000	Chr15:24899719..24902902	1088	361	6.18	40.82252	26.4	78.59	−0.465	cytoplasm
MdCYP27	MD15G1307700	Chr15:30716881..30721816	1785	594	8.24	64.79805	33.96	69.16	−0.486	nucleus
MdCYP28	MD16G1075600	Chr16:5293404..5295993	726	241	7.01	26.91272	48.35	75.27	−0.259	cytoplasm
MdCYP29	MD16G1123800	Chr16:8946852..8949641	780	259	9.54	27.53619	33.7	77.84	−0.173	chloroplast
MdCYP30	MD17G1286000	Chr17:34515787..34518173	762	253	8.52	27.86199	45.62	78.93	−0.094	chloroplast

*pI* Theoretical Isoelectric point, *MW* Molecular weight, *Ii* Instability Index, *Ai* Aliphatic index, *GRAVY* Grand average of hydropathicity

### The cyclophilin-like domain (CLD) of MdCYPs

**Fig. 2:**
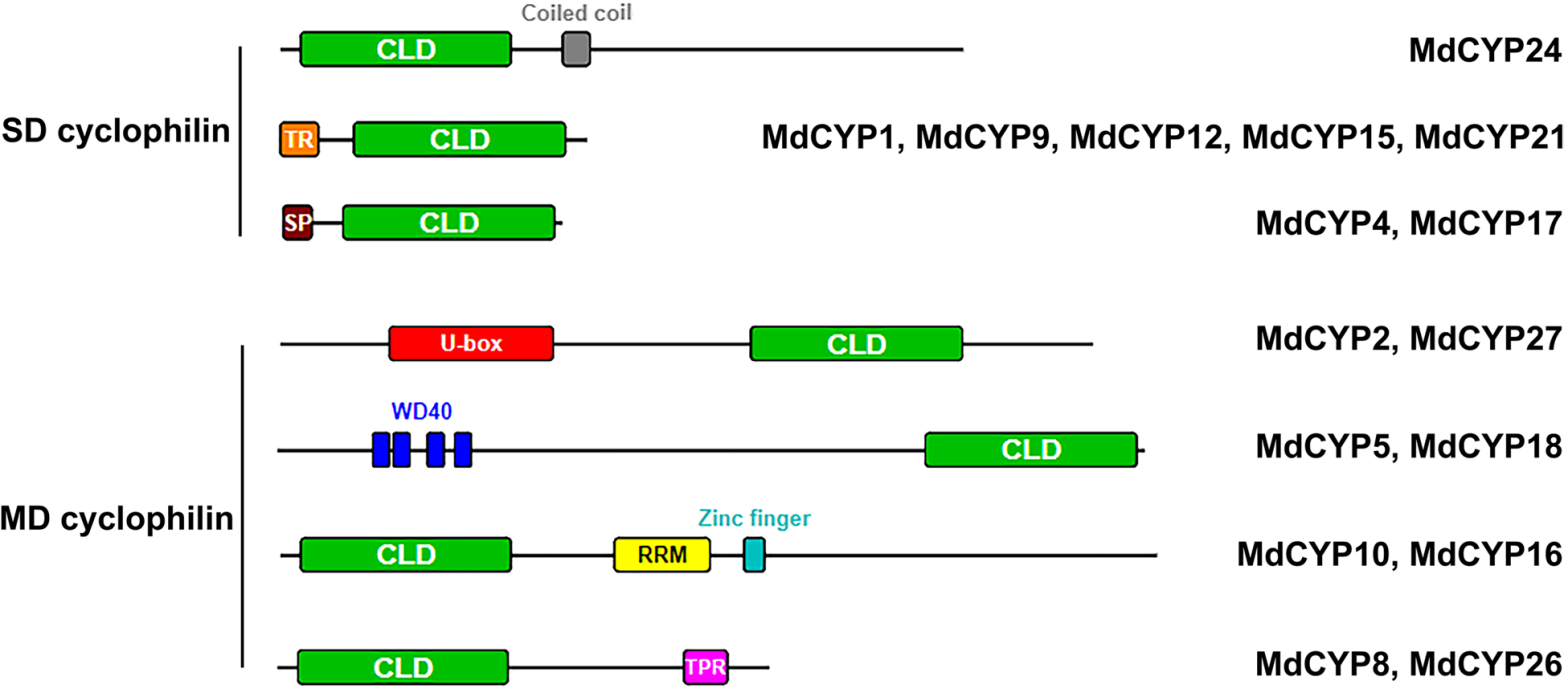
The single-domain (SD) cyclophilins and multi-domain (MD) cyclophilins in apple. Different color blocks represent different domains. Green: cyclophilin like domain (CLD); Black: coiled-coil region; Orange: transmembrane region (TR); Brown: signal peptides (SP); Red: U-box domain; Blue: WD-40 repeat domain; Yellow: RNA recognition motif (RRM); Cyan: zinc finger domain; Purplete: tratricopeptide repeat (TPR) domain

Multiple sequence alignment and amino acid conservation revealed the conserved nature of the CLD domain in the MdCYPs (Supplementary Fig. 1). Consistent with previous studies [11], MdCYPs could be divided into single-domain (SD) cyclophilins and multi-domain (MD) cyclophilins based on their CLD domains (Fig. 2). For the 22 SD CYPs, 10 MdCYPs have CLD domains located near the C-termini, six have CLD domains located in the middle of the coding sequence, and six have CLD domains located near the N-termini (Supplementary Fig. 2). Additionally, five MdCYPs contained a N-terminal transmembrane region (TR), two MdCYPs contained N-terminal signal peptides (SP), and MdCYP24 contained a coiled-coil region (Fig. 2). Among the eight MD CYPs, MdCYP2 and MdCYP27 both contain a U-box domain, MdCYP5 and MdCYP18 both contain a WD-40 repeat domain, MdCYP10 and MdCYP16 both contain an RNA recognition motif (RRM) and a zinc finger domain, and MdCYP8 and MdCYP26 both contain a tetratricopeptide repeat (TPR) domain (Fig. 2, Supplementary Fig. 2).

### Phylogenetic analysis of MdCYPs

**Fig. 3:**
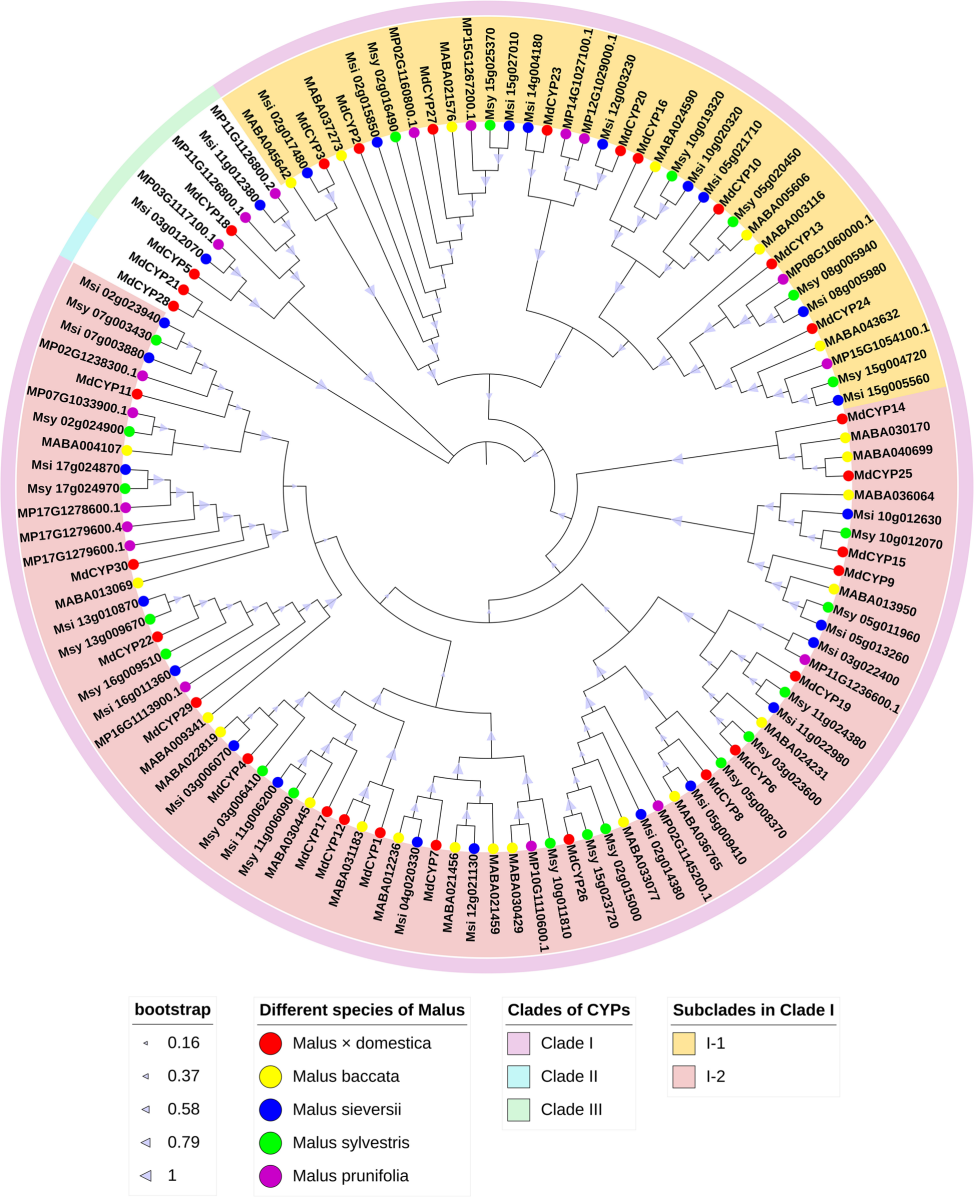
Phylogenetic analysis of MdCYPs in *Malus*. The 119 apple cyclophilin proteins were divided into three clades (the outer ring strips with different colors), and the different colored circles were used to distinguish the five apple species

Several genomes of the genus *Malus* have been published [43–46], and these have provided an important basis for systematic research. Here, we used the HMM strategy to search the cyclophilin family members of four other Malus species, *Malus sieversii*, *Malus sylvestris*, *Malus baccata*, and *Malus prunifolia*, and identified 26, 21, 24, and 18 cyclophilins, respectively (Supplementary Table 1). Subsequently, 119 cyclophilin protein sequences including 30 MdCYPs were used to construct a phylogenetic tree. The Phylogenetic tree was divided into three clades, CladeI included most of the cyclophilin members, and was subdivided into two sub-branches of CladeI-1 and CladeI-2. CladeIII included seven MD cyclophilins, including MdCYP5 and MdCYP18. CladeII contained only MdCYP21 and MdCYP28, which were independent from the other four species (Fig. 3).

### Gene structure, motif analysis, and 3D model of MdCYPs

**Fig. 4:**
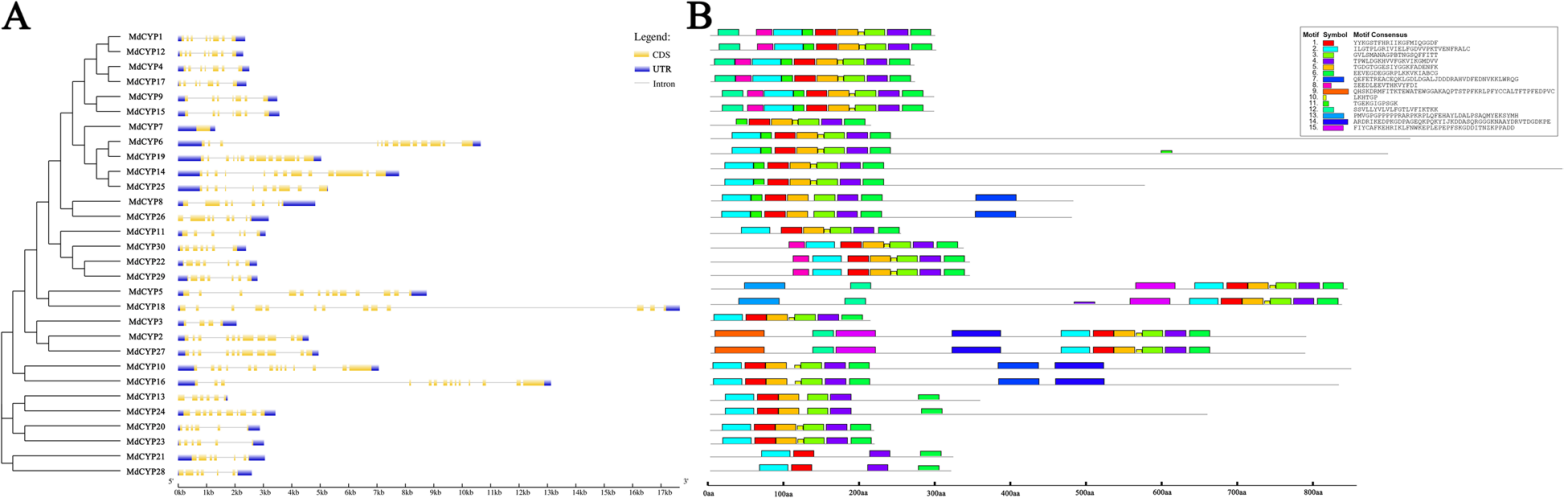
Gene structure (**A**) and motif analysis (**B**) of MdCYPs. The phylogenetic tree structure of MdCYPs was added on the left, and the amino acid sequences of 15 motifs were represented on the upper right

We interpreted the genome annotation of MdCYPs and analyzed the similarities and differences in the gene structure of this family. Overall, the total length of MdCYP genes ranged from 1500 to 18,000 bp, with MdCYP7 as the shortest gene and MdCYP18 as the longest gene. MdCYP7 contained only one exon, MdCYP10 and MdCYP16 contained the most exons, 14, with most MdCYPs containing six to eight exons (Fig. 4A). MEME analysis showed the distribution of 15 conserved motifs in these sequences. The analysis of the conserved amino acids of the identified motifs showed that the CLD domain was comprised of motif 1 to motif 6, and the other nine motifs are component units of TR, SP, U-box, TPR, WD-40 repeat, RRM, and zinc finger domains (Fig. 4B, Supplementary Fig. 3). The gene structure and motif analysis of MdCYPs with strong paralogy support the results of the evolutionary tree, and suggest that the proteins may play similar functions (Fig. 4).

**Fig. 5:**
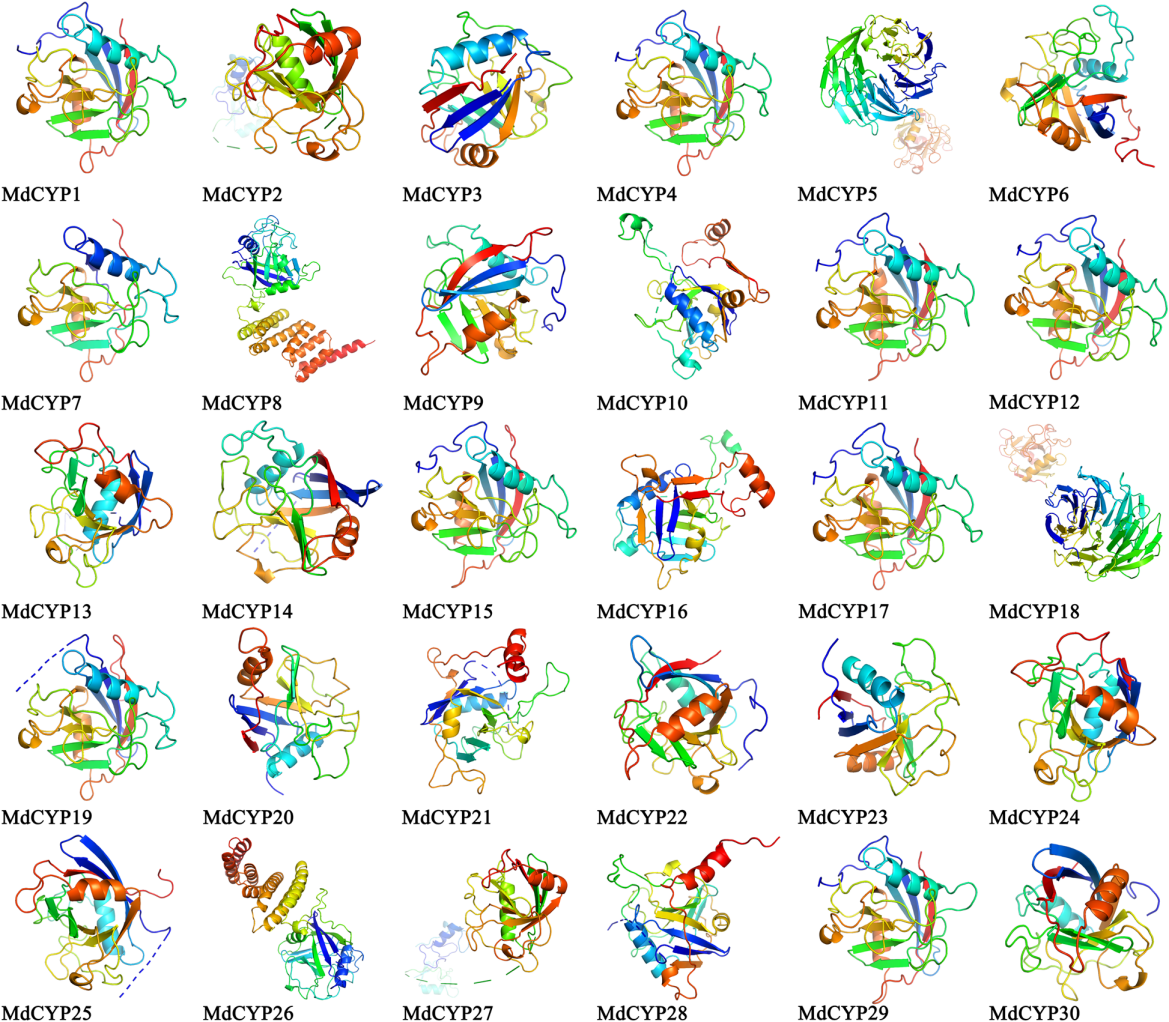
The three dimensional structure (3D) model of MdCYPs

The secondary structures of MdCYPs were predicted, and the proportions of the four secondary structures (alpha helices, beta turns, random coils, and extended strands) were analyzed for each CYP. Random coils accounted for the largest percentages in 28 MdCYPs, and only MdCYP8 and MdCYP26, two MD CYPs containing TPR domains, had a higher amount of alpha helices. MdCYP11 had the most beta turns, MdCYP14 had the most random coils, and MdCYP9 and MdCYP15 had the highest amount of extended strands (Supplementary Fig. 4). Subsequently, homologous modeling was applied to model the tertiary structure of the MdCYP proteins (Fig. 5, Supplementary Table 2). The 3D structural model shows that a typical CLD contains two alpha helices and eight antiparallel beta turns.

### Protein interaction network and *cis*-acting element analysis of MdCYPs

**Fig. 6:**
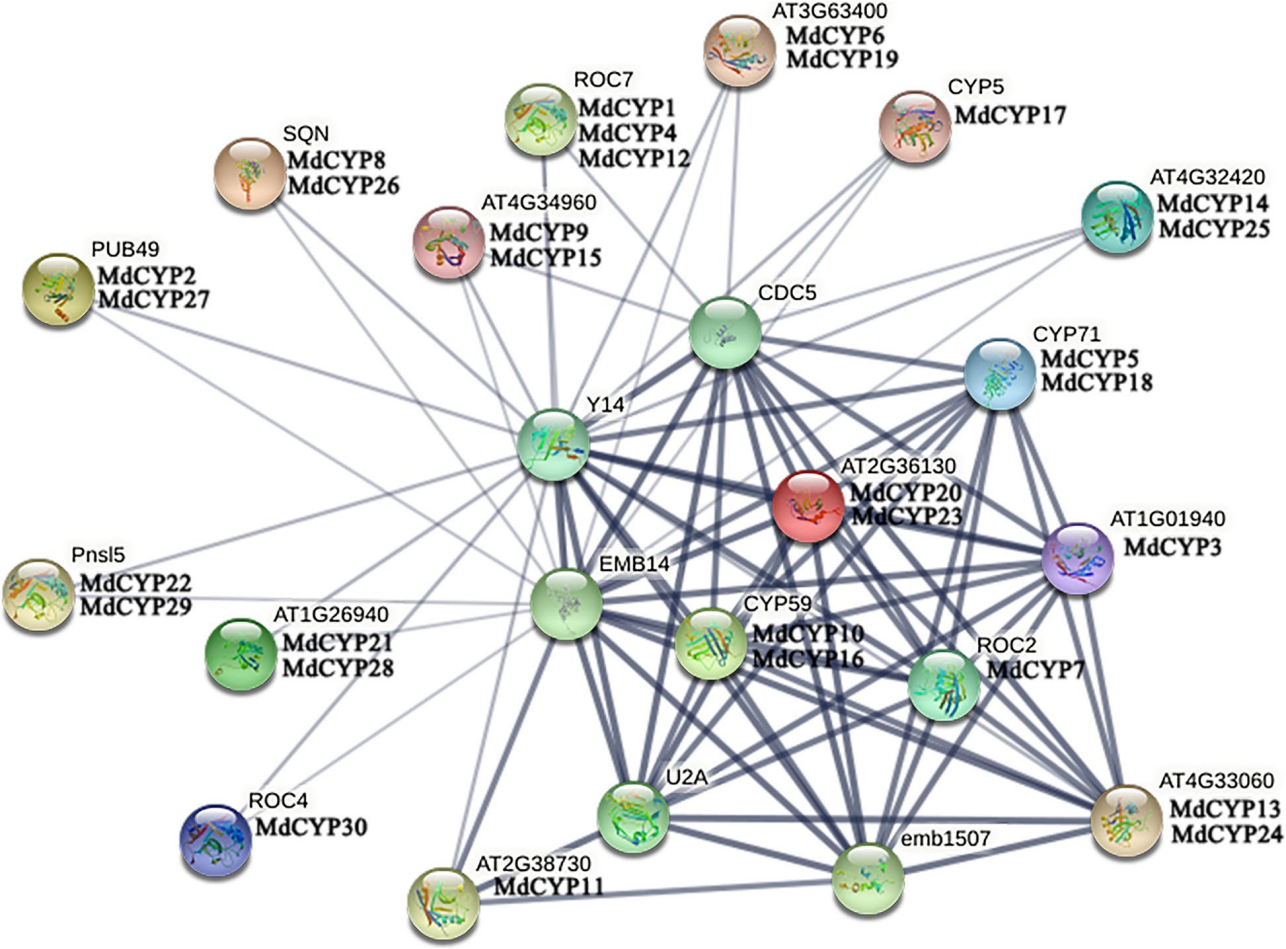
Protein interaction network of MdCYPs. Homologous CYP proteins in apple and *Arabidopsis*: AT1G01940:CYP18-1, AT2G36130:CYP18-2, ROC2:CYP19-3, CYP5:CYP19-4, ROC7:CYP20-1, Pnsl5:CYP20-2, ROC4:CYP20-3, AT4G34960:CYP21-1, AT2G38730:CYP22-1, AT1G26940:CYP23-1, SQN:CYP40, AT4G33060:CYP57, CYP59:CYP59, AT3G63400:CYP63, PUB49:CYP65, CYP71:CYP71, AT4G32420:CYP95. The proteins that interact with the MdCYP proteins: CDC5: cell division cycle 5-like protein, Y14: RNA binding (RRM/RBD/RNP motifs) family protein, EMB14: Pre-mRNA-processing-splicing factor, U2A: U2 small nuclear ribonucleoprotein A, emb1507: U5 small nuclear ribonucleoprotein helicase

To predict the potential function of CYPs in apple, a interaction network of MdCYP proteins was constructed using orthologous sequences from the model species *Arabidopsis* (Fig. 6). The MdCYPs corresponded to the 17 AtCYPs, and these proteins interact with small nuclear ribonucleoprotein, small nuclear ribonucleoprotein helicase, Pre-mRNA-processing-splicing factor, RNA binding (RRM/RBD/RNP motifs) family protein, and cell division cycle 5-like protein. According to the complexity of the network, MdCYP3, MdCYP5, MdCYP7, MdCYP10, MdCYP13, MdCYP16, MdCYP18, MdCYP20, MdCYP23, and MdCYP24 were associated with more proteins, suggesting more important roles.

**Fig. 7:**
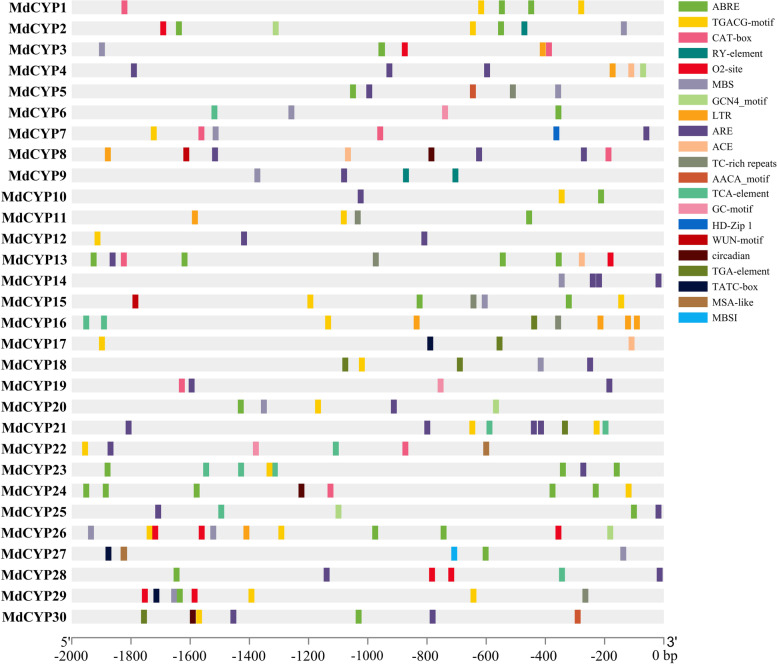
The *cis*-element analysis of MdCYPs. ABRE: cis-acting element involved in the abscisic acid responsiveness. TGACG-motif: cis-acting regulatory element involved in the MeJA-responsiveness. CAT-box: cis-acting regulatory element related to meristem expression. RY-element: cis-acting regulatory element involved in seed-specific regulation. O2-site: cis-acting regulatory element involved in zein metabolism regulation. MBS: MYB binding site involved in drought-inducibility. GCN4_motif: cis-regulatory element involved in endosperm expression. LTR: cis-acting element involved in low-temperature responsiveness. ARE: cis-acting regulatory element essential for the anaerobic induction. ACE: cis-acting element involved in light responsiveness. TC-rich repeats: cis-acting element involved in defense and stress responsiveness. AACA_motif: involved in endosperm-specific negative expression. TCA-element: cis-acting element involved in salicylic acid responsiveness. GC-motif: enhancer-like element involved in anoxic specific inducibility. HD-Zip 1: element involved in differentiation of the palisade mesophyll cells. WUN-motif: wound-responsive element. Circadian: cis-acting regulatory element involved in circadian control. TGA-element: cis-acting element involved in auxin responsiveness. TATC-box: cis-acting element involved in gibberellin-responsiveness. MSA-like: cis-acting element involved in cell cycle regulation. MBSI: MYB binding site involved in flavonoid biosynthetic genes regulation

The promoter sequences 2000 base pairs upstream from the ATG start codon were collected, and the *cis*-acting elements of all MdCYPs were analyzed and visualized (Fig. 7). The promoters of MdCYPs contained multiple *cis*-acting elements, including elements previously shown to be involved in growth and development processes (meristem growth, seed-specific regulation, endosperm development, circadian control, and cell cycle regulation), elements that respond to phytohormones (auxin, gibberellin, abscisic acid, jasmonate and salicylic acid), and elements that respond to environmental stress (light, low temperature, and drought). The MeJA-responsiveness TGACG-motif was identified in the promoters of 17 MdCYPs, suggesting a link to jasmonate signaling.

### Collinearity analysis of *MdCYPs*

**Fig. 8:**
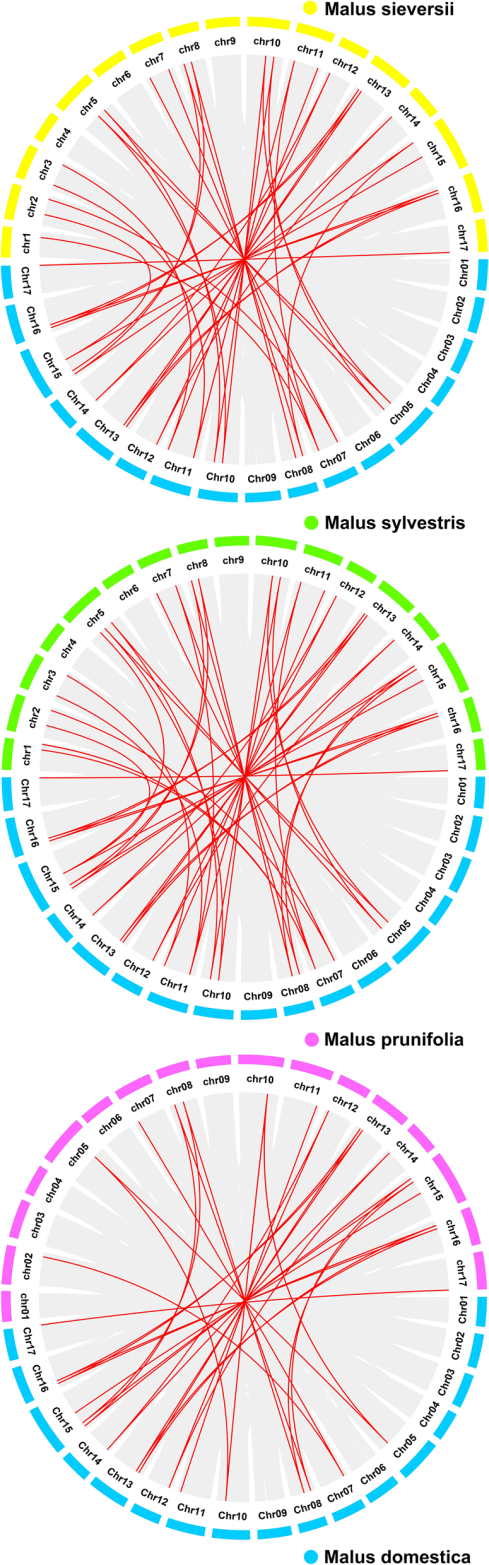
Collinearity analysis of *MdCYPs* among *Malus*. Duplication events within *M. domestica* and the genomes of the other three *Malus* species (*M. sieversii*, *M. sylvestris* and *M. prunifolia*) are indicated with grey lines in the background, while the red lines highlight syntenic CYP gene pairs

To investigate the mechanism of gene duplication events in the CYP family, we performed a genome-wide collinearity analysis in *M. domestica* (Supplementary Fig. 5). This approach can identify historical events in the number and composition of chromosomes. The results showed that 17 chromosomes are derived from a putative nine-chromosome ancestor. Each doublet of the eight apple chromosomes (3/11 and 13/16) is derived principally from ancient chromosomes II and IV, respectively. Chromosomes 1/7 originate from duplications of ancient chromosomes VII and IX, followed by a translocation and a deletion event. Similar events generated chromosomes 8/15 from chromosomes VIII and IX [47]. Among the 17 collinear gene pairs identified, according to the traditional definitions of segmental duplication and tandem duplication, a total of ten pairs of segmental duplication genes were identified, but no tandem duplication genes were found (Supplementary Table 3). We next performed collinearity analysis among the four *Malus* genomes, and 38, 43, and 29 intergenomic duplications were identified between *M. domestica* and *M. sieversii*, *M. sylvestris,* and *M. prunifolia*, respectively (Fig. 8, Supplementary Table 4).

### Analysis of tissue expression patterns of *MdCYPs*

**Fig. 9:**
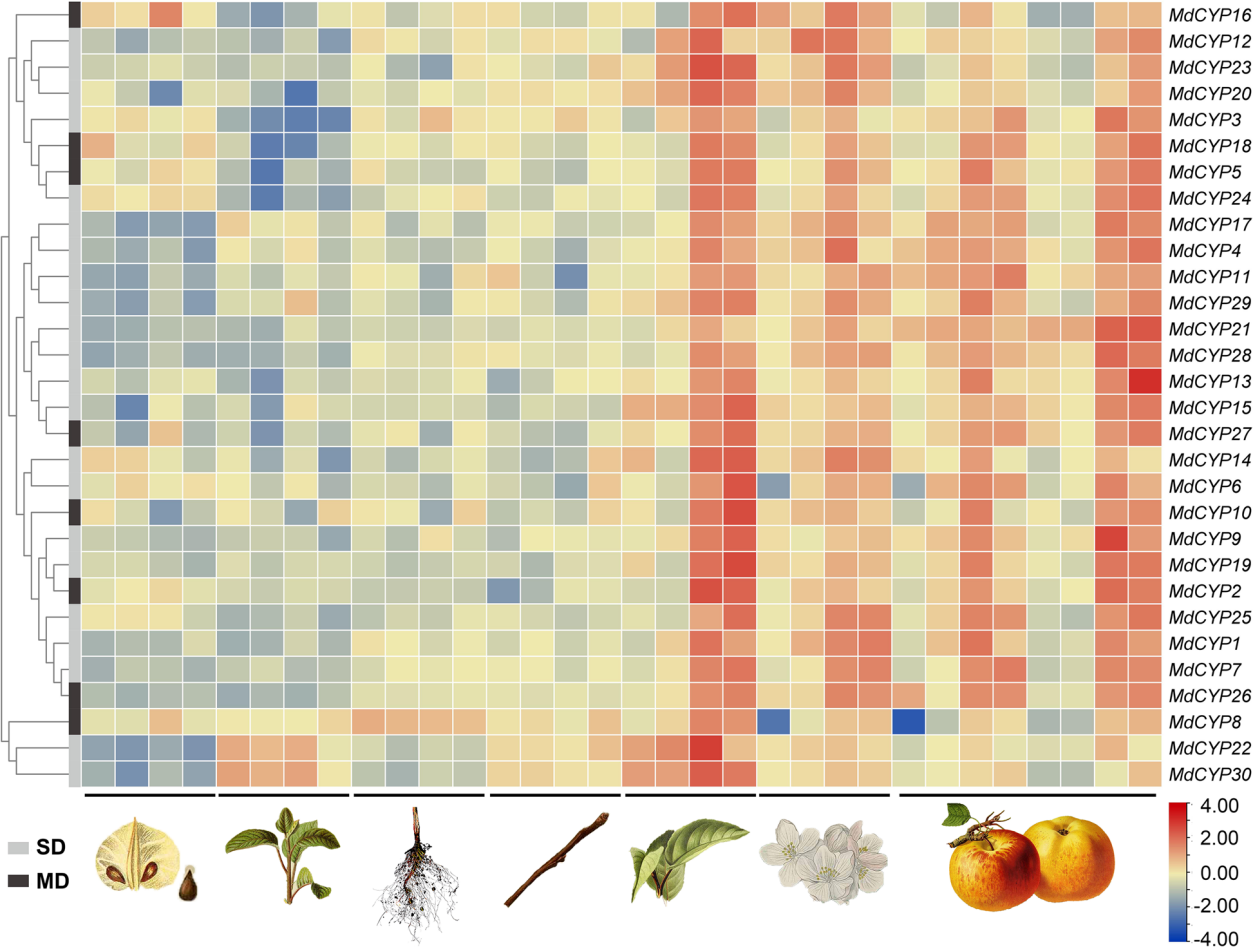
DNA Microarray data of *MdCYPs* in different organs. The tree structure on the left represents the cluster analysis of data, and the graph on the bottom represents the different organs of the apple: seed, seedling, root, stem, leaf, flower and fruit. SD: Single Domain Cyclophilin, MD: Multidomain Cyclophilin

Tissue-specific or biased expression of genes often is related to gene function. Next, we analyzed and clustered the tissue expression pattern data of CYPs (Fig. 9). Overall, the 30 MdCYP genes were highly expressed in leaves, flowers, and fruit organs. However, there was variation in expression for different CYPs. For example, MdCYP16, MdCYP 18, and MdCYP24 were highly expressed in seeds, MdCYP8 was highly expressed in roots, and MdCYP22 and MdCYP 30 were more highly in seedlings than other MdCYPs. Tissue-specific expression was not shown previously for SD or MD CYPs.

### Expression patterns analysis of *MdCYPs* under abiotic stress

**Fig. 10:**
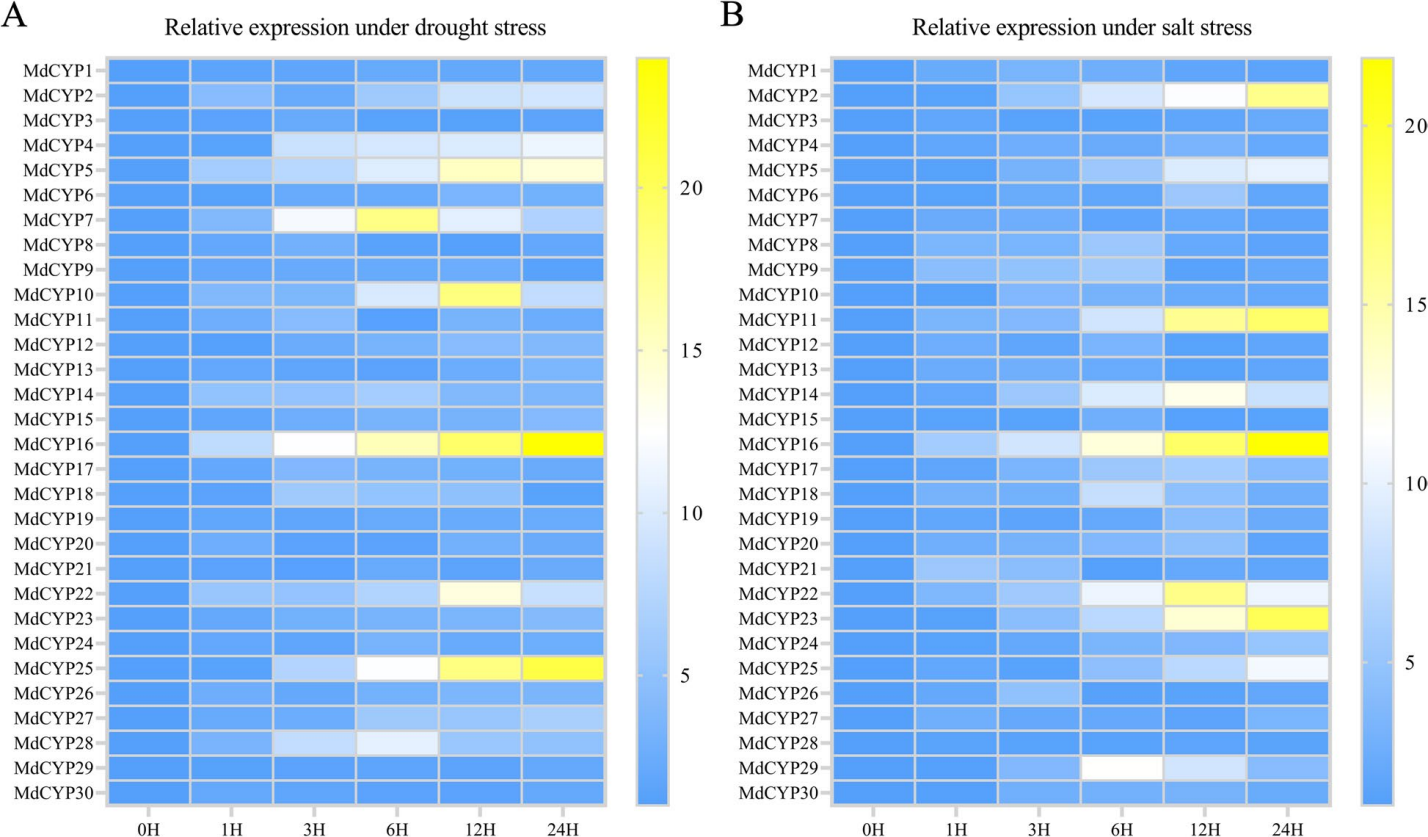
The qRT-PCR profile of *MdCYPs* under drought stress simulated by 6% PEG6000 (**A**) and salt stress simulated by 150 mM NaCl (**B**). Samples were taken in different time periods (0 h, 1 h, 3 h, 6 h, 12 h and 24 h) after treatment. The data were normalized to the apple *MdActin* expression level, each experiment involved three biological repetitions

Multiple *CYP*s have been reported to be involved in various abiotic stresses. Next, we examined the expression changes of *MdCYPs* under different abiotic stresses (drought stress simulated by 6% PEG6000 or salt stress simulated by 150 mM NaCl) by qRT-PCR. The results of drought treatment showed up-regulated expression of ten *MdCYPs*, with *MdCYP5*, *MdCYP7*, *MdCYP10*, *MdCYP16*, *MdCYP22,* and *MdCYP25* significantly up-regulated at different times after treatment; the highest was 24-fold higher than the untreated control (Fig. 10A). In addition, eight *MdCYPs* responded to salt stress. *MdCYP11*, *MdCYP16*, *MdCYP22,* and *MdCYP23* were significantly up-regulated at different times after treatment, with the highest 21-fold higher than the untreated control (Fig. 10B). In aggregate, expression was significantly up-regulated 12 h and 24 h after treatment. These results suggest that MdCYPs are responsive to drought and salt stress and mediate plant stress response. Notably, *MdCYP16* exhibited a change in expression in response to both drought stress and salt stress (Fig. 10).

### MdCYP16 is a key nuclear localization cyclophilin involved in abiotic stress

**Fig. 11:**
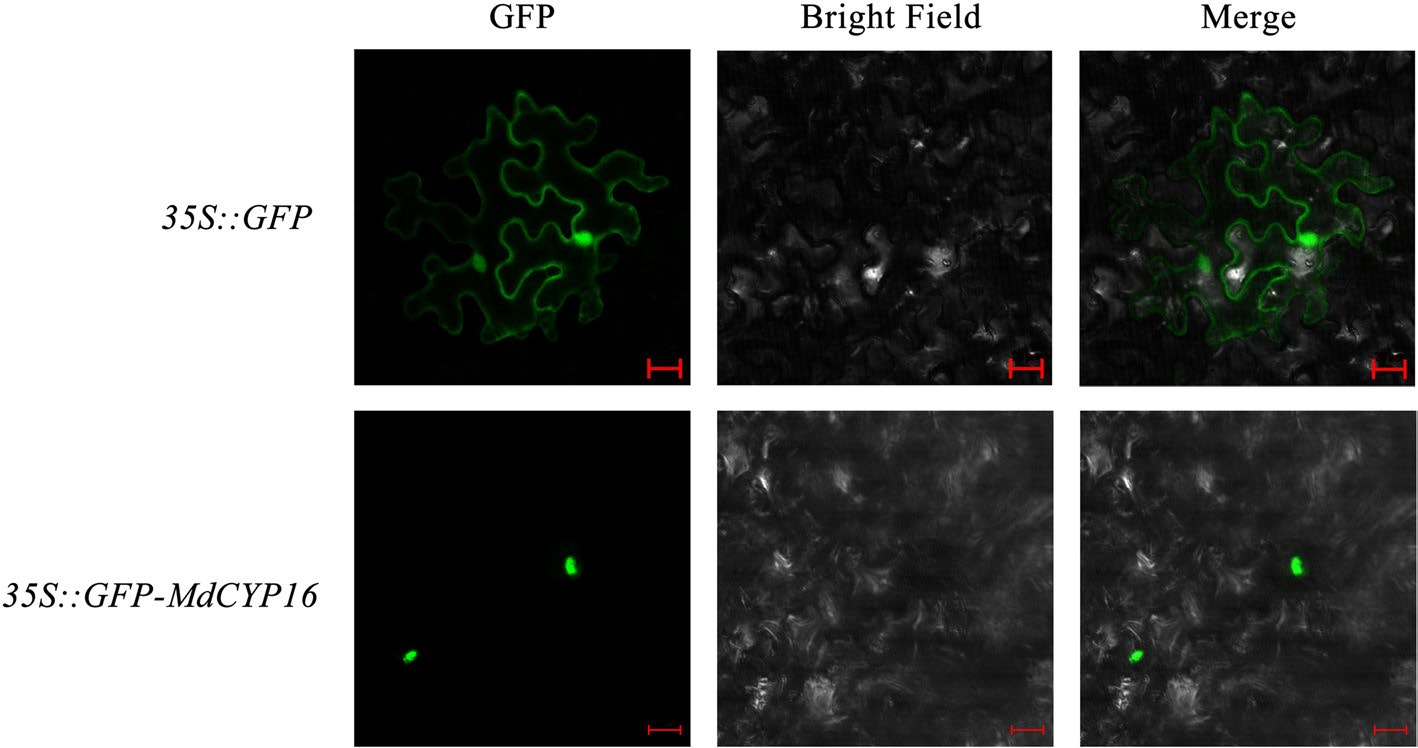
Subcellular localization of the MdCYP16. Fusion proteins *35S::GFP-MdCYP16* were observed in the nucleus of *N.benthamiana* leaf epidermal cells by laser confocal microscopy. The *35S::GFP* was used as a control (scale bar, 20 μm)

To verify the results of qRT-PCR, we further investigated the expression of *MdCYP16*. MdCYP16 contains the RRM and zinc finger domains, and MdCYP16 was predicted to be located in the nucleus (Table 1). To confirm this, we cloned the full-length CDS of *MdCYP16* from apple, and ligated this DNA into the pRI-101 AN vector to add a green fluorescent protein (GFP) tag (Supplementary Table 5). The localization of fusion protein 35S::GFP-MdCYP16 was observed by transient transformation of *Nicotiana benthamiana* leaves. Green fluorescence driven by strong promoters was observed throughout the epidermal cells, whereas green fluorescence of MdCYP16-GFP fusion protein was only be observed in the nucleus, confirming localization of MdCYP16 to the nucleus (Fig. 11).

**Fig. 12:**
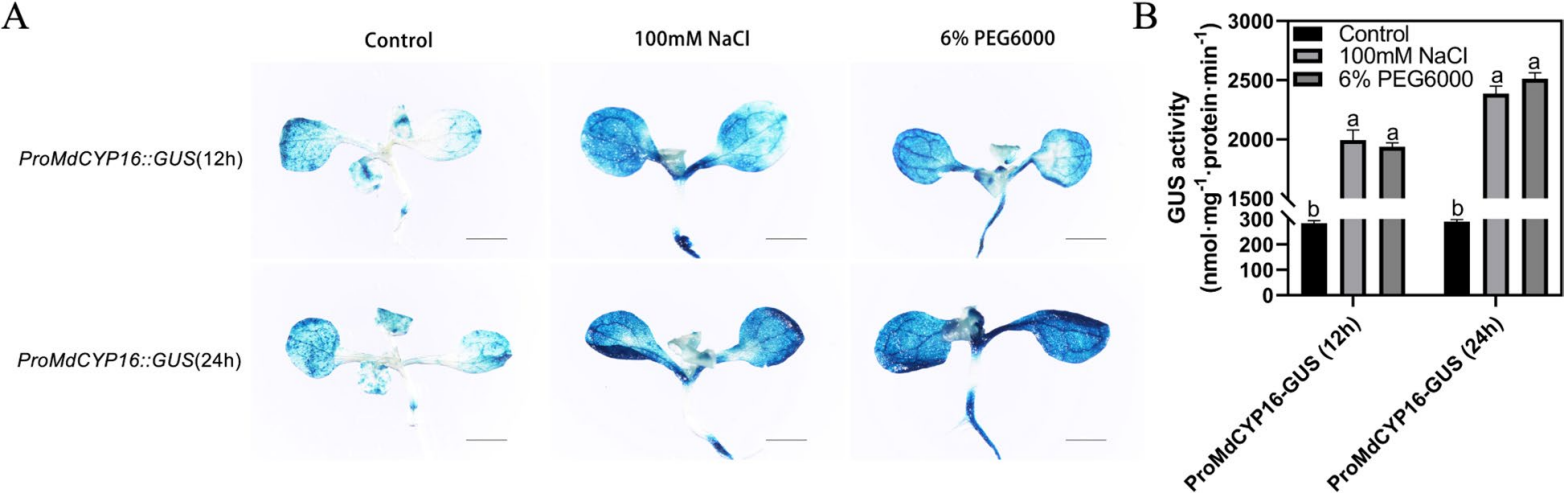
GUS staining in *ProMdCYP16::GUS* transgenic *Arabidopsis*. **A** Phenotype of *ProMdCYP16::GUS* transgenic *Arabidopsis* under untreated conditions, drought stress simulated by 6% PEG6000 and salt stress simulated by 150 mM NaCl at 12 h and 24 h (scale bar, 1000 μm). **B** Statistics of GUS activity detection of *ProMdCYP16::GUS* transgenic *Arabidopsis.* Each experiment involved three biological repetitions. Different letters represent significant differences (ANOVA, Turkey correction, *p* < 0.05)

We engineered *ProMdCYP16::GUS* transgenic *Arabidopsis*, performed the same abiotic stress treatment, and then detected the expression of *MdCYP16* using GUS staining. The GUS staining of transgenic *Arabidopsis* did not change with time without treatment. However, after 100 mM NaCl treatment for 12 h, the color of leaves became darker, and the color of leaves was deepest after 24 h treatment (Fig. 12A). Similar results were observed under PEG treatment, and were supported by quantitative analysis of GUS activity (Fig. 12B). These data indicated that expression of *MdCYP16* was sensitive to salt and drought stress, which was consistent with the qRT-PCR results (Fig. 10). Together, these results suggest that *MdCYP16* acts in salt and drought resistance.

## Discussion

At least seven CYP families have been identified and the number of *CYPs* in model plants and food crops range from a minimum of 28 to a maximum of 94 [11–17]. However, little work has focused on these proteins in horticultural crops. Here, we performed a systematic genome-wide analysis of the apple cyclophilin gene family and identified 30 CYP members from the most recent ‘Golden Delicious’ apple genome. The sequences all contained a conserved CLD domain and included single-domain SD CYPs and mutli-domain MD CYPs (Table 1, Fig. 2).

Of the SD MdCYPs, the sequences can be subdivided into three categories: only contain CLD, contain N-terminal TR and CLD, and contain N-terminal SP and CLD (Fig. 2, Supplementary Fig. 2). The first two categories are the same as described in analysis of these proteins in *Arabidopsis* [11], but we did not find a C-terminal TR domain similar to *AtCYP26-1* in apple. In addition, the TR identified in cotton cyclophilin GbCYP142 is present in the middle region of MD CYPs, not in SD CYPs [48]. Notably, SD CYPs containing SP have not been described in *Arabidopsis*, but our analysis predicts that MdCYP4 and MdCYP17 localize to extracellular and vacuoles, respectively (Fig. 3), which may be directly related to their SP activity. Studies in rice found that OsCYP20-2, a typical SD CYP, changes its localization from the chloroplast to the nucleus through alternative splicing to exert different functions [40]. These findings suggest the importance of SD cyclophilin N-terminal TR and SP domains in subcellular localization.

For MD, MdCYPs can be divided into four classes based on shared CLD domains (Fig. 2). This classification is consistent with the four classes of ten GmCYPs (TPR of GmCYP8, GmCYP9, GmCYP16 and GmCYP17, U-box of GmCYP18 and GmCYP19, WD-40 repeat of GmCYP20, GmCYP35, and RRM and zinc finger domain GmCYP56, GmCYP59) identified in soybean [13]. However, MD CYPs of *Arabidopsis* and oilseed rape contain other domains including Leu zipper and Ser/Lys-Arg/Glu-rich regions that have not been found in apple [11, 15]. Interestingly, four of the eight MD CYPs (AtCYP38, AtCYP59, AtCYP63 and AtCYP95) in *Arabidopsis* are located in the nucleus, while all the apple MD CYPs (MdCYP2, MdCYP5, MdCYP6, MdCYP10, MdCYP13, MdCYP14, MdCYP16, MdCYP19, MdCYP25 and MdCYP27) are located in the nucleus (Table 1). The CYP families previously identified in maize, rice, alfalfa, and other species do not completely correspond to the classification of MD CYPs in *Arabidopsis* [12, 14, 17], and some of them have evolved new domains including Herpes ICP4 (infected-cell poly-peptide 4) C-domain (MtCYP95A and MtCYP95B) and Rho domains (MtCYP59A and MtCYP59B). These differences indicate the complexity and diversity of cyclophilin families, suggesting different functions in the evolution of different plant species.

In the phylogenetic tree analysis, given that both MdCYP5 and MdCYP18 of CladeIII contain WD domains, we guessed that the remaining five cyclophilins from *M. prunifolia* and *M. sieversii* in CladeIII also contain WD domains. We performed domain checks on the remaining cyclophilins in CladeIII, and the results were consistent with this speculation (Supplementary Fig. 6). This indicates that the MD CYP containing the WD domain diverged from other members of the CYP family, suggesting different functions. CladeII and CladeIII do not contain CYP from *M. baccata* (Siberia in northern Asia) or wild apple *M. sylvestris* in Europe [49] (Fig. 3). This result is not surprising since cultivated apple *M. domestica*, Xinjiang wild apple *M. sieversii,* and *M. prunifolia* are geographically close together [49, 50]. As the origin of cultivated apple, *M. sieversii* from the Tianshan Mountains was used for genetic exchanges with different species of *Malus* during the Silk Road period, and was finally domesticated as the popular hybrid cultivated apple *M. domestica* [51, 52]. The native species *M. prunifolia* is a species of crabapple that is widely distributed in China and has been cultivated for more than 2000 years for the selection of excellent rootstock. *M. prunifolia* may be a hybrid between *M. baccata* and *M. sieversii* in Kazakhstan [43, 50]. The grouping into CladeII and CladeIII suggests that *M. domestica*, *M .sieversii*, and *M. prunifolia* retain specialized functions in Central and East Asia, and the proteins encoded by these cyclophilin genes may be conserved overall but not shared with other *Malus* species.

Gene duplication is a major driving forces of genome and genetic system evolution, and segmental duplication and tandem duplication are the two main causes of gene family expansion in plants [53]. Segmental duplications can result in multiple genes through polyploidy followed by chromosome rearrangements, and this process occurs easily in plants because most plants are diploidized polyploids [54]. In tandem duplications, multiple members of one family may be within the same intergenic region or in neighboring intergenic regions, with no more than one intervening gene [55]. Here, we did not find tandemly duplicated CYP gene pairs, but found segmental duplications (Supplementary Fig. 4). Based on the evolutionary history of the golden crown apple genome, these segmental duplications all occurred with the chromosomal doubling rearrangement event [47]. The gene structures and protein structures of tandemly duplicated CYP gene pairs are very similar to each other (Figs. 4 and 5). In conclusion, large-scale whole-genome duplication events (mainly segmental duplications) are the main driving force for the expansion of apple CYP family. The interspecific collinearity analysis revealed a relative large number of gene pairs between *M. domestica* and *M .sieversii* and *M. sylvestris,* with the latter two, as wild apples in Asia and Europe, contributing the main gene composition to the cultivated apple *M. domestica*. Overall, the results of collinearity among the three species were roughly similar, indicating the commonality of CYP in *Malus* (Fig. 8).

The expression of *CYPs* occurs in all organs of the plants. *GmCYP*s are highly expressed in young tissues of soybean [13]. *SsCYP* is highly abundant in transporting organs, tubers, open flowers, and stamens of *Solanaceae*, but the abundance in leaves strongly decreases with age [56]. Overall, our analysis of apple expression profile found highest expression of *MdCYPs* in leaves, followed by reproductive organs, flowers, and fruits (Fig. 9). Previous studies identified a cyclophilin in the pollen of higher plants including lily and *Nicotiana tabacum*, that can be released into the extracellular matrix under unfavorable conditions to regulate the elongation and orientation of pollen tubes [57]. These results all indicate that cyclophilin expression can vary in different developmental stages, with differences in protein abundances during the developmental process.

Abiotic stresses such as extreme temperature, drought, and high salinity are important environmental factors that restrict plant growth and development [58]. Currently, cyclophilin proteins in *Arabidopsis* and rice have been identified that can effectively help plants cope with the challenges of environmental stress. *AtROC3* (*CYP19-1*) and *AtROC1* (*CYP18-3*) of *Arabidopsis* positively regulate drought response and freezing tolerance, respectively [41, 42]. Rice *OsCYP2* and *OsCYP21-4* positively regulate salt stress and confer salt tolerance [35, 38]. Rice *OsCYP20-2* responds to osmotic stress and then low temperature stress [37, 40]. In our results, ten and eight *MdCYPs* were found to respond to drought and salt stress, respectively, with induced expression levels after treatment (Fig. 10). The *MdCYP16* responded to both drought and salt stress, and is predicted to be a nuclear-localized MD CYP (Fig. 11), which was not reported previously. The orthologous gene in *Arabidopsis* is *AtCYP59*, which was reported to be involved in the processing of pre-mRNA during transcriptional regulation [26]. Interestingly, overexpression of *OsCYP18-2* in transgenic rice and *Arabidopsis* enhanced drought resistance and altered the expression of stress-related genes and pre-mRNA splicing patterns in *Arabidopsis* under drought conditions [39]. This led us to speculate that *MdCYP16* may be involved in a similar regulatory network, but it is involved in abiotic stress as an MD CYP. We confirmed the evidence that this CYP responds to drought and salt stress in *ProMdCYP16::GUS* transgenic *Arabidopsis* by GUS staining (Fig. 12). Therefore, the results show that *MdCYP16* is an important member of the cyclophilin family in apple.

## Conclusions

In this study, 30 cyclophilin genes on 15 chromosomes were systematically identified in the apple (*M. domestica*) genome. Phylogenetic analysis showed that the cyclophilin family members are divided into three clades in *Malus*. Paralogous genes generated by segmental duplication facilitated the expansion of the apple cyclophilin gene family. Analysis of the gene and protein structure further supported the results of the phylogenetic tree and collinearity analysis. The expression of MdCYPs was higher in leaves, flowers, and fruits. Ten and eight CYPs responded to drought and salt stress, respectively. MdCYP16, a nuclear-localized MD CYP, was found to be highly sensitive to drought and salt stress and GUS staining results of transgenic *Arabidopsis* indicated that *MdCYP16* responds to abiotic stress. Overall, our study provides important insight into cyclophilins in horticultural crops such as apple, and provides valuable information for further analysis of the function of CYP genes to guide breeding efforts for improved apple stress resistance.

## Methods

### Plant materials and growth condition

‘Royal Gala’ (*M. domestica*) and ‘Columbia’ *Arabidopsis* were used in this study. ‘Royal Gala’ tissue-cultured apple plantlets were planted on Murashige-Skoog (MS) medium containing 0.2 mg/L naphthyl acetic acid (NAA), 0.5 mg/L gibberellin acid (GA), and 1.0 mg/L 6-benzylaminopurine (6-BA) at 24 °C under long-day conditions (16 h light/8 h dark). The *Arabidopsis* seedlings seeds were surface-sterilized and germinated on 1/2 MS nutrient medium. After 3-5 days of growth, seedlings were transferred to an artificial growth chamber at 22 °C under a 16 h-light/8 h-dark photoperiod [59].

### Identification and characterization of cyclophilin family of *Malus*

The genome databases of five species of the genus *Malus* (*M. domestica*, *M. sieversii*, *M. sylvestris*, *M. baccata* and *M. prunifolia*) were from the Genome Database for Rosaceae (GDR, https://www.rosaceae.org/). Based on the query strategy of Hidden Markov Model, the file of cyclophilin protein conserved domain CLD (PF00160) was downloaded from the Pfam database (http://pfam.xfam.org/). Next, the hmmsearch command was executed to search for CYPs in all apple proteins, where E-value was set to less than 1e^− 5^. The corresponding protein sequence was extracted using the scanned sequence number, and the CLD domain was checked again on the Simple Molecular Architecture Research Tool (SMART, http://smart.embl-heidelberg.de/), and the confirmed members were candidate MdCYPs [60].

### Statistical analysis of physicochemical properties

The physical and chemical characteristics, inluding molecular weight size, isoelectric point and other information of MdCYP proteins were calculated using the online tool ProtParam (http://web.expasy.org/protparam) in ExPASY. The secondary structure composition was predicted on SOPMA (https://npsa-prabi.ibcp.fr/cgi-bin/npsa_automat.pl?page=npsa_sopma.html), and on Phyre2 (http://www.sbg.bio.ic.ac.uk/phyre2/html/page.cgi?id=index), the three-dimensional (3D) structure homology modeling was performed, [61].

### Chromosome localization and multiple sequence alignment

The position information of MdCYP family members were extracted according to the gene ID in the apple genome annotation file, and submitted to the online software MG2C (http://mg2c.iask.in/mg2c_v2.0/) for gene chromosome distribution. Protein multi-sequence alignment was performed on Clustal Omega (https://www.ebi.ac.uk/Tools/msa/clustalo/) [62], and the alignment results were edited and visualized in the desktop Jalview 2.10.5 [63].

### Phylogenetic tree construction and subcellular localization prediction

All CYP proteins were used to construct phylogenetic trees in Molecular Evolutionary Genetics Analysis (MEGA7) software [64], and Muscle program was built into the multi-sequence alignment with the default parameters, neighbor-joining method was selected for phylogenetic tree, bootstrap method was selected for the test and repeated 1000 times, and poisson model was selected for the replacement model, and iTOL (https://itol.embl.de/personal_page.cgi) as a beautification tool for phylogenetic trees. Subcellular localization was predicted using the online program WoLF PSORT (https://www.genscript.com/wolf-psort.html), biological type selection of plants.

### Gene structure and conserved motif analysis

The gene characteristics of MdCYPs were analyzed in Peking University Gene Structure Visualization Server GSDS2.0 (http://gsds.cbi.pku.edu.cn/). The conservative motif analysis was performed using the Multiple Em for Motif Elucidation (MEME5.1.1, https://meme-suite.org/meme/tools/meme).

### Protein homology modeling analysis

All MdCYP protein sequences were submitted to the Phyre2 (http://www.sbg.bio.ic.ac.uk/phyre2/html/page.cgi?id=index) for homology modeling. The template corresponding to each CYP was obtained from the PDB (https://www.rcsb.org/) database, and the Root Mean Square Deviation RMSD value was calculated [65].

### Gene function prediction and protein interaction network

Functional protein association network online website STRING (https://string-db.org) was used to predict the interactions of proteins related to members of the CYP family, and species source selection model species *Arabidopsis thaliana*.

### The cis-acting element analysis of promoter

The 2000 bp fragment upstream of ATG, the translation initiation site, was extracted from the apple genome as the promoter region, and all the MdCYP promoter sequences were submitted to PlantCare (http://bioinformatics.psb.ugent.be/webtools/plantcare/html/) for the predictive analysis of the cis acting elements of the promoter, and the results were visualized in TBtools software [66].

### Collinearity analysis within and between species

The genome sequence file and annotated file of apple and *Arabidopsis* from GDR and TAIR (https://www.arabidopsis.org/), respectively. Run MCScanX in TBtools software, and the collinear gene pairs within apple species and between apple and *Arabidopsis* species were obtained under the whole gene background.

### Analysis of tissue expression pattern

CYP gene expression data were from Array Express (https://www.ebi.ac.uk/arrayexpress/), select E-GEOD-42873 microarray data set (‘X4442X2596’ ‘X3069X922’ ‘Golden Delicious’ ‘X4102’ ‘X8877’ ‘M14’ ‘M49’ ‘M67’ ‘M74’ ‘M20’ and other apple resources). The number in the microarray was converted and matched using the ‘Golden Delicious’ Apple accession number. All data were sorted out and normalized (Z-Score model).

### Plasmid construction and genetic transformation

The open reading frame (ORF) of *MdCYP16* was cloned into the pENTR/D/TOPO vector (Invitrogen), and a green fluorescent protein (GFP) was fused to MdCYP16 to obtain *35S::MdCYP16-GFP* construct. The construct was transformed into *A. tumefaciens* LBA4404 and infiltrated into *Nicotiana benthamiana* leaves. The promoter sequence (2000 bp length) upstream of the *MdCYP16* initiation codon was cloned and inserted into the pCAMBIA1300 vector containing the GUS reporter gene to generate a *ProMdCYP16::GUS* construct. The fusion construct was transformed into *Arabidopsis* using the floral dip transformation method [67]. The above sequences are collated in the Supplementary Table 3.

### Subcellular localization and microscopy

The empty plasmid *35S::GFP* was transformed into *A. tumefaciens* LBA4404 and infiltrated into *Nicotiana benthamiana* leaves as the control. After transient infection, the tobacco was placed in darkness overnight and then grew for 2 to 3 days in a 16 h-light/8 h-dark photoperiod. The lower epidermis of the leaves were torn off for a laser scanning confocal microscopic observation.

### Abiotic stress treatment

For the drought stress and salt stress treatment, consistent and healthy ‘Royal Gala’ tissue-cultured apple plantlets were treated with 6% PEG6000 and 100 mM NaCl for 1 week under a 16 h/8 h light/dark photoperiod at 25 °C, respectively. The 5-day-old *Arabidopsis* seedlings were treated with 6% PEG6000 and 100 mM NaCl for 7 days under a 16 h/8 h light/dark photoperiod in a culture room at 23 °C, respectively.

### RNA extraction and quantitative real-time PCR’ (qRT PCR) analysis

Total RNAs of apple plantlets and *Arabidopsis* seedlings were isolated using an RNA extraction kit (Tiangen, Beijing, China) and used for qRT-PCR analysis following previously described methods [68]. Three technical and biological replicates were performed to detect the transcripts of MdCYPs gene. The MdActin (GenBank accession number CN938024) was used as the internal control. Quantitative analysis of gene expression was performed based on the 2^-∆∆CT^ method [69]. Specific primers of the MdCYPs gene were designed and listed in Supplementary Table 4.

### GUS (β-Glucuronidase) histochemical staining

The 12 day *ProMdCYP16::GUS* transgenic *Arabidopsis* after stress treatment were stained in a GUS staining buffer (containing 0.5 mM ferrocyanide, 0.1% Triton X-100, 0.1 mM EDTA, 0.5 mM ferricyanide and 1 mM X-Gluc), and then were decolorized with absolute ethyl alcohol for 12 h [70]. The GUS staining process was not repeated.

### Statistical analysis

Three biological repetitions, each containing three technical replicates, were performed. DPS software was used for data analysis, and differences were considered statistically significant when *p* < 0.05 using Tukey’s single-factor tests. Data are shown as mean ± standard deviation (SD).

## Supplementary Information

**Additional file 1: Supplementary Figure 1.** Multiple sequence alignment and amino acid conservation of MdCYP proteins. **Supplementary Figure 2.** All protein domains of MdCYPs. **Supplementary Figure 3.** Amino acid sequences and conservation of 15 motifs. **Supplementary Figure 4.** Secondary structure statistics of MdCYP proteins. **Supplementary Figure 5.** Collinearity analysis of *MdCYPs* in the apple genome-wide context. **Supplementary Figure 6.** Protein domains of 5 CYPs in CladeIII. **Supplementary Table 1.** Information of CYPs in *Malus* (*M. sieversii*, *M. sylvestris*, *M. baccata* and *M. prunifolia*). **Supplementary Table 2.** Information statistics of MdCYP family protein homology modeling. **Supplementary Table 3.** Segmental duplication genes in colinear gene pairs of *MdCYPs*. **Supplementary Table 4.** The intergenomic duplications between. **Supplementary Table 5.** The qRT-PCR primer sequences of *MdCYPs*. **Supplementary Table 6.** Primer of coding sequence and promoter sequence of *MdCYP16*.

## Data Availability

All data generated or analysed during this study are included in this article [and its supplementary information files]. The datasets generated in this study are available from the NCBI GEO database under accession number GSE214087. The accession numbers of all apple original sequences and *Arabidopsis* original sequences are stored in the Genome Database for Rosaceae (GDR, https://www.rosaceae.org/) and Arabidopsis Information Resource (TAIR, https://www.arabidopsis.org/), respectively.

## Abbreviations

CYP: Cyclophilin
CLD: Cyclophilin-like domain
SD: Single-domain
MD: Multi-domain
MW: Molecular weight
TR: Transmembrane region
SP: Signal peptides
RRM: RNA recognition motif
TPR: Tetratricopeptide repeat
GFP: Green fluorescent protein
GUS: β-Glucuronidase
ROS: Reactive oxygen species
qRT-PCR: Quantitative Real-time Polymerase chain reaction
