# Supplementary material for: Genome-wide identification and stress response analysis of cyclophilin gene family in apple (Malus × domestica)
Source: BMC Genomics. 2022 Dec 6;23:806. doi: 10.1186/s12864-022-08976-w (PMC9727951; doi:10.1186/s12864-022-08976-w)
Supplement: Supplementary file 1 — Additional file 1: Supplementary Figure 1. Multiple sequence alignment and amino acid conservation of MdCYP proteins. Supplementary Figure 2. All protein domains of MdCYPs. Supplementary Figure 3. Amino acid sequences and conservation of 15 motifs. Supplementary Figure 4. Secondary structure statistics of MdCYP proteins. Supplementary Figure 5. Collinearity analysis of MdCYPs in the apple genome-wide context. Supplementary Figure 6. Protein domains of 5 CYPs in CladeIII. Supplementary Table 1. Information of CYPs in Malus (M. sieversii, M. sylvestris, M. baccata and M. prunifolia). Supplementary Table 2. Information statistics of MdCYP family protein homology modeling. Supplementary Table 3. Segmental duplication genes in colinear gene pairs of MdCYPs. Supplementary Table 4. The intergenomic duplications between. Supplementary Table 5. The qRT-PCR primer sequences of MdCYPs. Supplementary Table 6. Primer of coding sequence and promoter sequence of MdCYP16. [file 12864_2022_8976_MOESM1_ESM.docx]

**Supplementary Figure 1**

Multiple sequence alignment and amino acid conservation of MdCYP proteins.


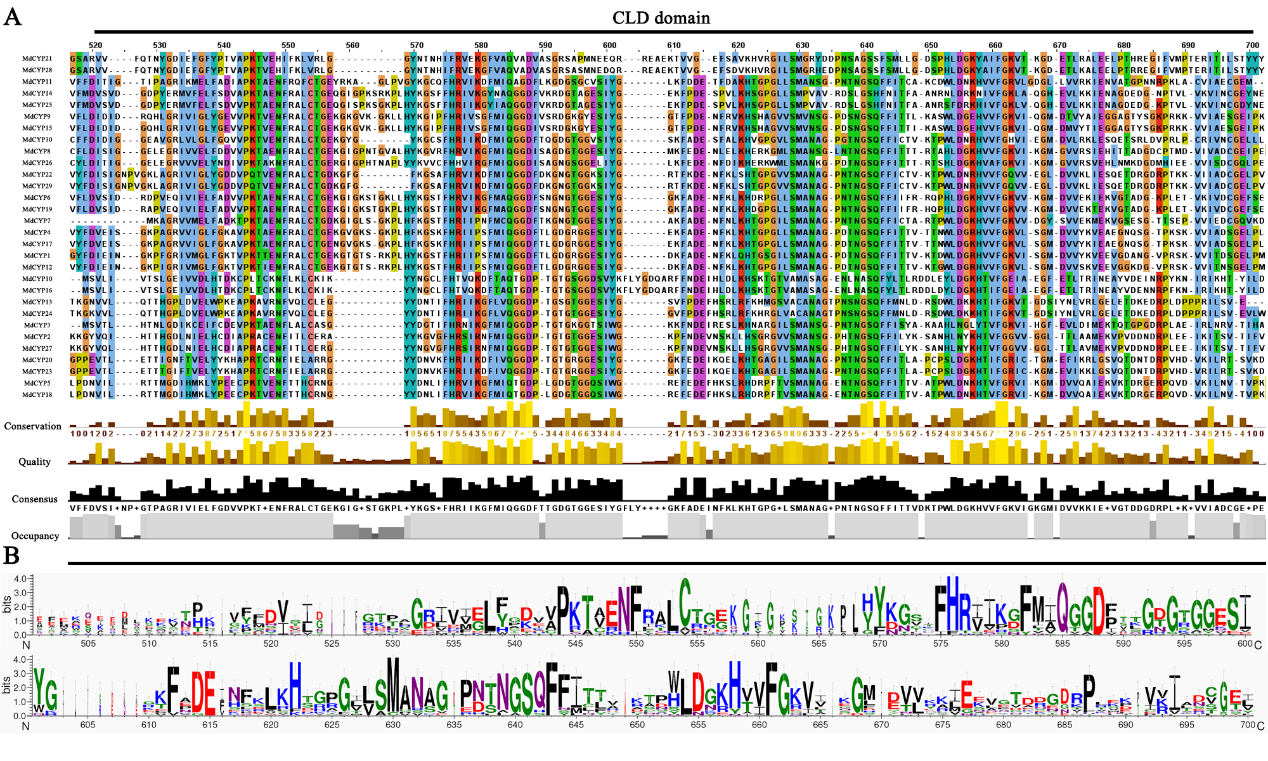


**Supplementary Figure 2**

All protein domains of MdCYPs


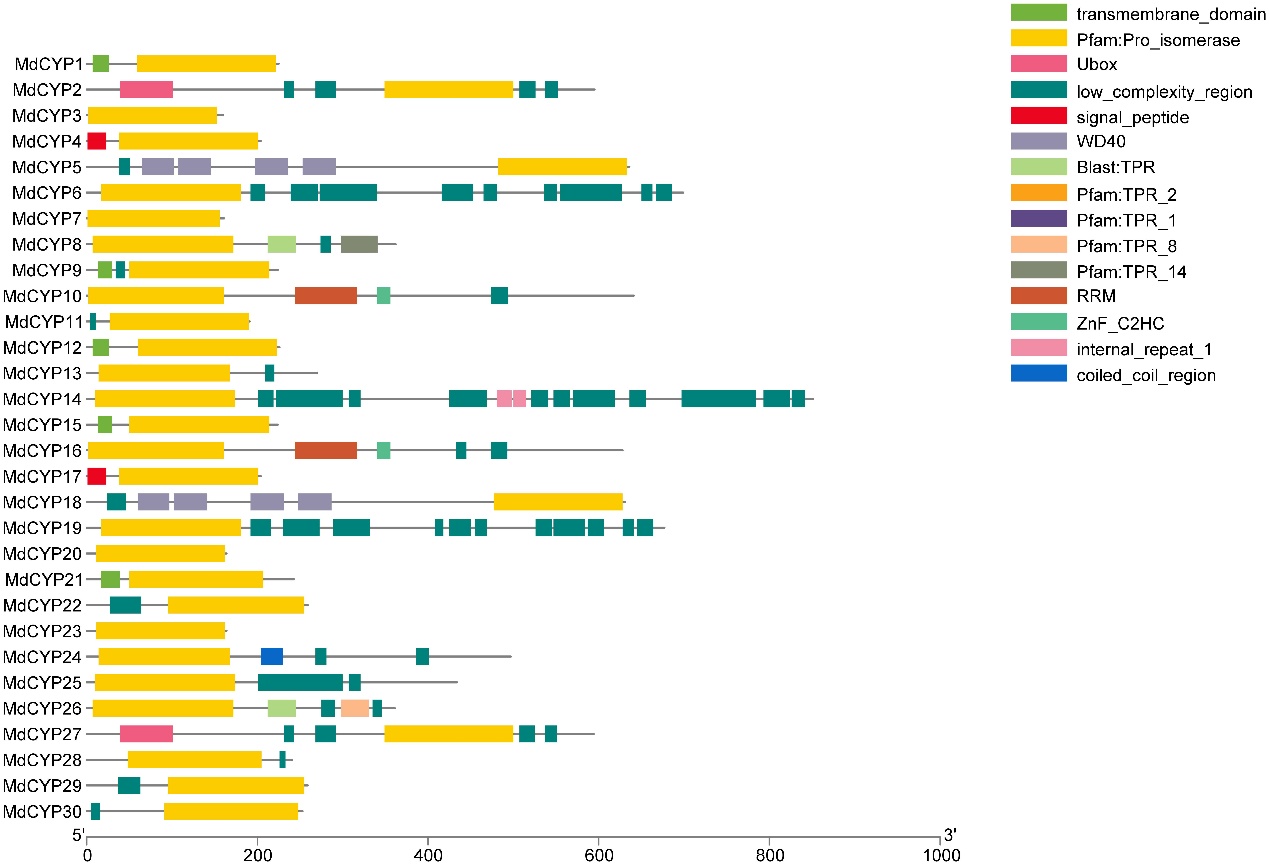


**Supplementary Figure 3**

Amino acid sequences and conservation of 15 motifs.


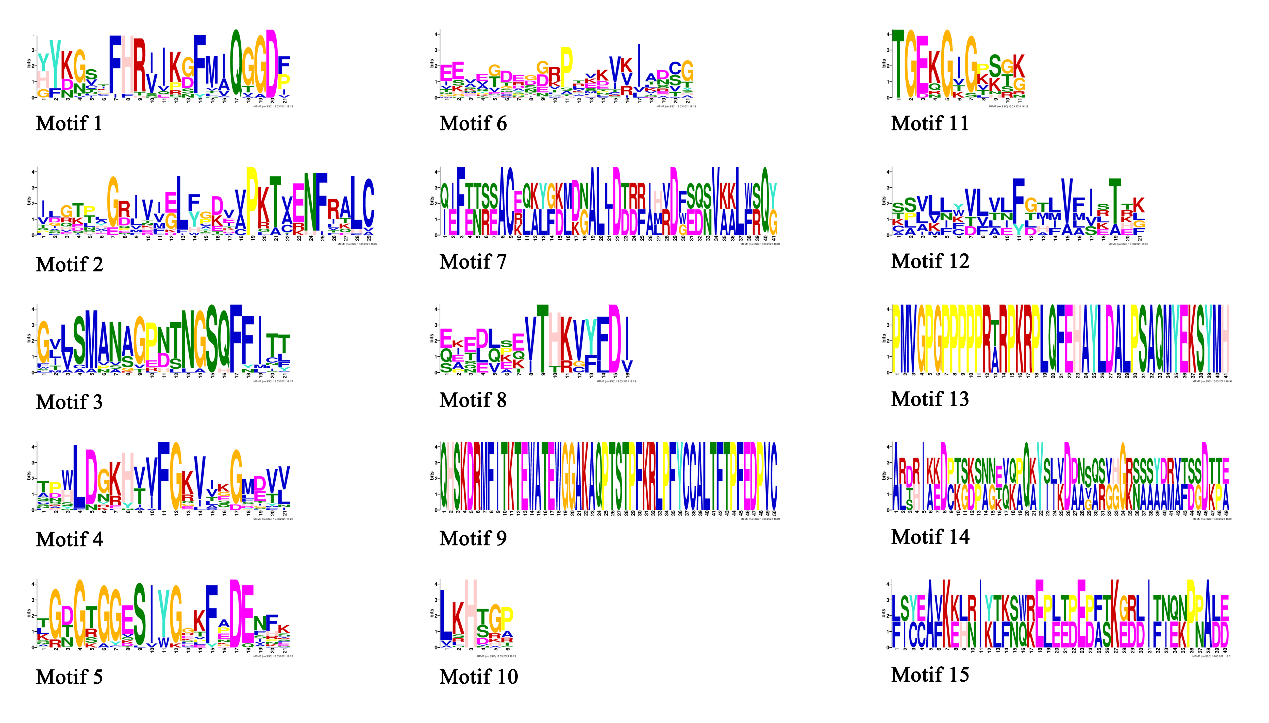


**Supplementary Figure 4**

Secondary structure statistics of MdCYP proteins.


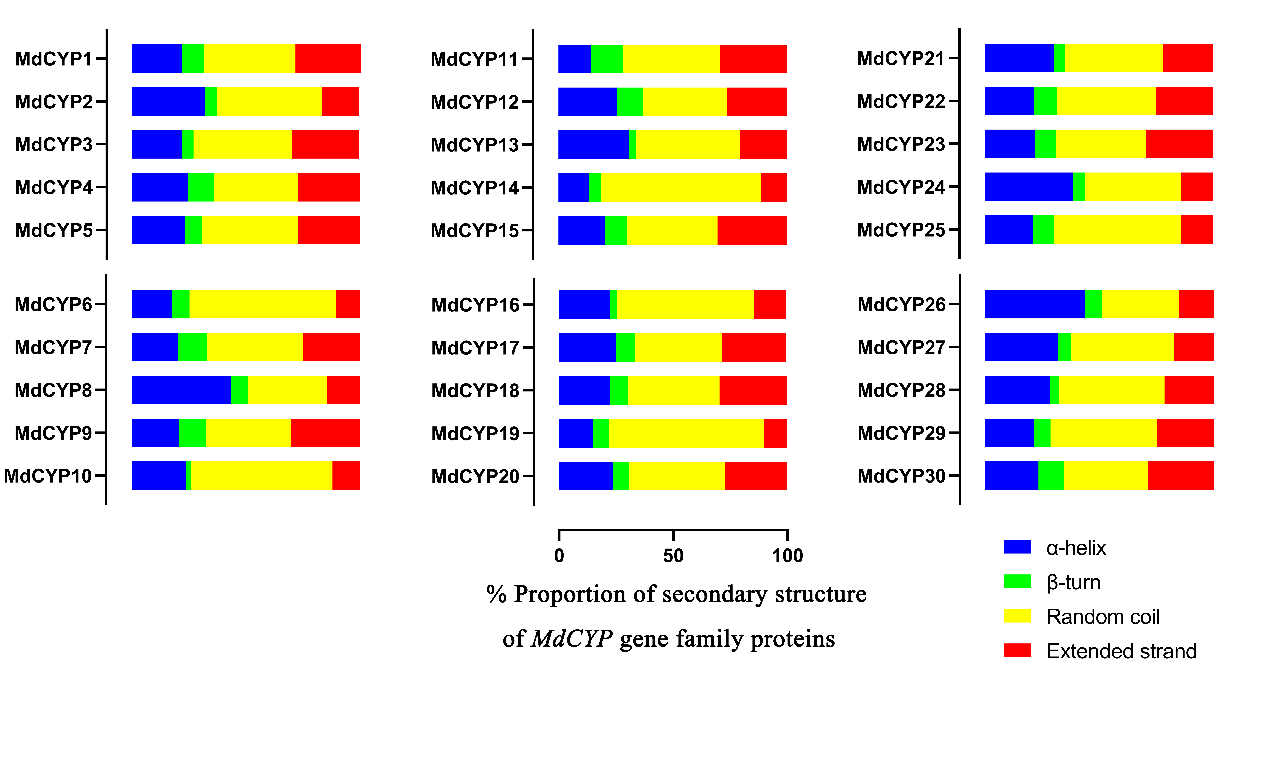


**Supplementary Figure 5**

Collinearity analysis of *MdCYPs* in the apple genome-wide context.


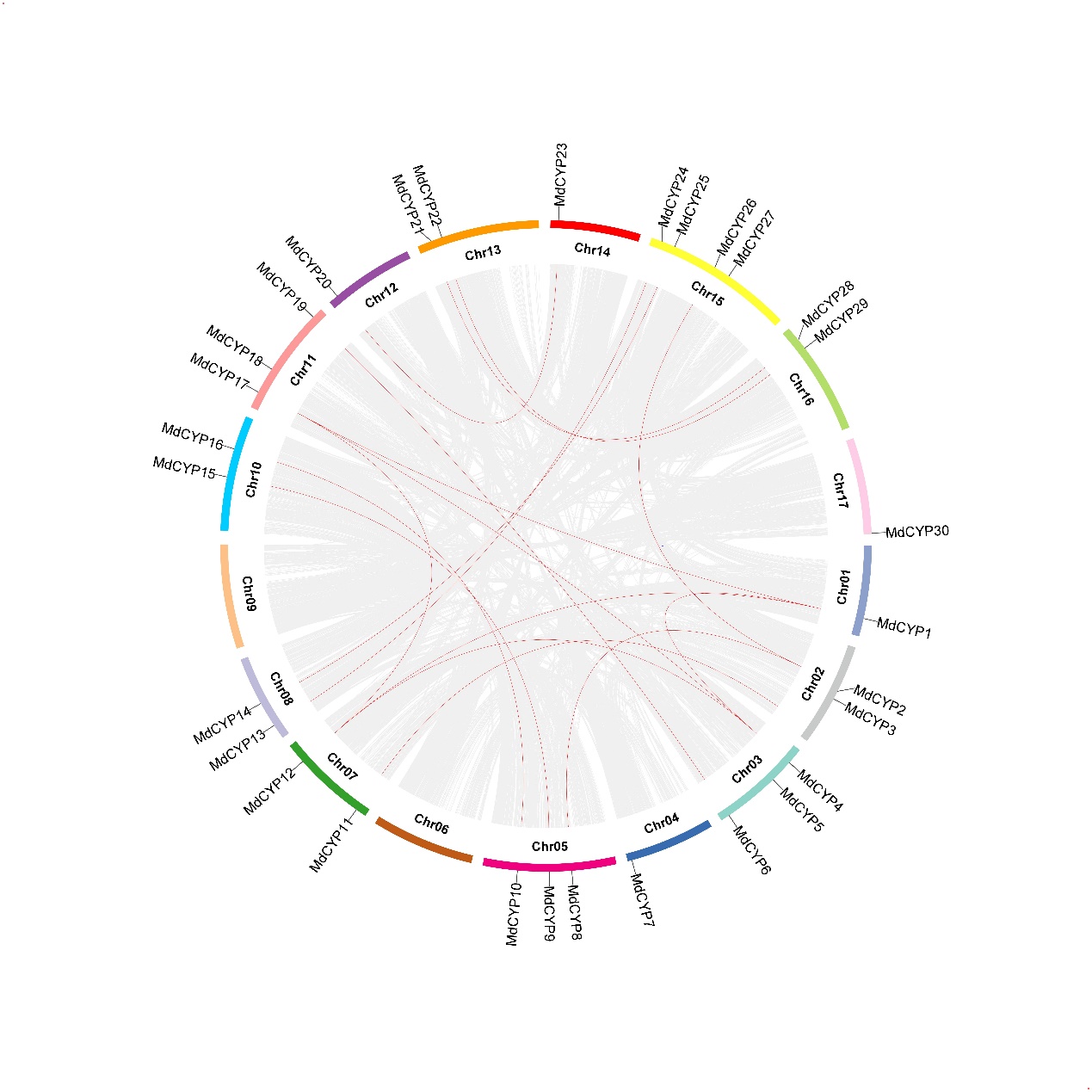


**Supplementary Figure 6**

Protein domains of 5 CYPs in CladeIII


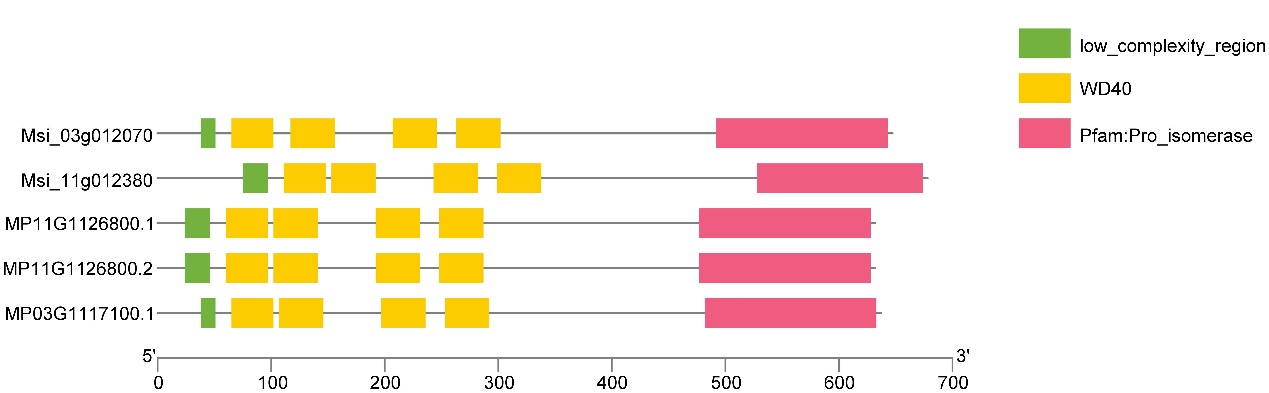


**Supplementary Table 1**

Information of CYPs in *Malus* (*M. sieversii*, *M. sylvestris*, *M. baccata* and *M. prunifolia*)

| ***M. baccata*** (24) | ***M. sieversii*** (26) | ***M. sylvestris*** (21) | ***M. prunifolia*** (18) |
| --- | --- | --- | --- |
| MABA003116 | Msi_16g011360 | Msy_15g023720 | MP16G1113900.1 |
| MABA004107 | Msi_17g024870 | Msy_05g011960 | MP12G1029000.1 |
| MABA005606 | Msi_14g004180 | Msy_13g009670 | MP08G1060000.1 |
| MABA009341 | Msi_02g014380 | Msy_15g025370 | MP02G1238300.1 |
| MABA012236 | Msi_02g017480 | Msy_10g011810 | MP02G1160800.1 |
| MABA013069 | Msi_04g020330 | Msy_02g016490 | MP02G1145200.1 |
| MABA013950 | Msi_03g006070 | Msy_11g024380 | MP10G1110600.1 |
| MABA021456 | Msi_15g005560 | Msy_17g024970 | MP11G1236600.1 |
| MABA021459 | Msi_03g012070 | Msy_03g023600 | MP11G1126800.1 |
| MABA021576 | Msi_07g003880 | Msy_11g006090 | MP11G1126800.2 |
| MABA022819 | Msi_02g023940 | Msy_07g003430 | MP07G1033900.1 |
| MABA024231 | Msi_15g027010 | Msy_08g005940 | MP03G1117100.1 |
| MABA024590 | Msi_05g021710 | Msy_02g015000 | MP17G1279600.1 |
| MABA030170 | Msi_10g012630 | Msy_02g024900 | MP17G1279600.4 |
| MABA030429 | Msi_10g020320 | Msy_16g009510 | MP17G1278600.1 |
| MABA030445 | Msi_03g022400 | Msy_05g008370 | MP14G1027100.1 |
| MABA031183 | Msi_05g009410 | Msy_15g004720 | MP15G1054100.1 |
| MABA033077 | Msi_13g010870 | Msy_03g006410 | MP15G1267200.1 |
| MABA036064 | Msi_12g021130 | Msy_05g020450 |  |
| MABA036765 | Msi_08g005980 | Msy_10g012070 |  |
| MABA037273 | Msi_12g003230 | Msy_10g019320 |  |
| MABA040699 | Msi_11g012380 |  |  |
| MABA043632 | Msi_11g006200 |  |  |
| MABA045642 | Msi_05g013260 |  |  |
|  | Msi_11g022980 |  |  |
|  | Msi_02g015850 |  |  |

**Supplementary Table 2**

Information statistics of MdCYP family protein homology modeling.

The second column is the number of the template protein, which is included in the PDB (https://www.rcsb.org/). The third column is **R**oot **M**ean **S**quare **D**eviation.

| **Protein** | **Template** | **RMSD** |
| --- | --- | --- |
| MdCYP1 | 2IGV | 0.283 |
| MdCYP2 | 7DVQ | 7.445 |
| MdCYP3 | 2OK3 | 0 |
| MdCYP4 | 2IGV | 0.285 |
| MdCYP5 | 7A5P | 0 |
| MdCYP6 | 1IHG | 0 |
| MdCYP7 | 2IGV | 0.298 |
| MdCYP8 | 1IHG | 0 |
| MdCYP9 | 2RMC | 0.463 |
| MdCYP10 | 2K7N | 1.341 |
| MdCYP11 | 1QOI | 0.401 |
| MdCYP12 | 2IGV | 0.285 |
| MdCYP13 | 2HQ6 | 0.674 |
| MdCYP14 | 1IHG | 0 |
| MdCYP15 | 2ESL | 0.457 |
| MdCYP16 | 2K7N | 1.342 |
| MdCYP17 | 2IGV | 0.283 |
| MdCYP18 | 7A5P | 0 |
| MdCYP19 | 1IHG | 0 |
| MdCYP20 | 1XWN | 1.821 |
| MdCYP21 | 5EX2 | 0.717 |
| MdCYP22 | 7EU3 | 0.618 |
| MdCYP23 | 1XWN | 1.821 |
| MdCYP24 | 2HQ6 | 0.697 |
| MdCYP25 | 1IHG | 0 |
| MdCYP26 | 1IHG | 0 |
| MdCYP27 | 7DVQ | 7.445 |
| MdCYP28 | 5EX1 | 0.185 |
| MdCYP29 | 7EU3 | 0.618 |
| MdCYP30 | 7EU3 | 0.623 |

**Supplementary Table 3**

Segmental duplication genes in colinear gene pairs of *MdCYPs*.

| Gene localization | Gene name | Gene localization | Gene name |
| --- | --- | --- | --- |
| Chr1 | MdCYP1 | Chr7 | MdCYP12 |
| Chr3 | MdCYP4 | Chr11 | MdCYP17 |
| Chr3 | MdCYP5 | Chr11 | MdCYP18 |
| Chr3 | MdCYP6 | Chr11 | MdCYP19 |
| Chr5 | MdCYP9 | Chr10 | MdCYP15 |
| Chr5 | MdCYP10 | Chr10 | MdCYP16 |
| Chr8 | MdCYP13 | Chr15 | MdCYP24 |
| Chr8 | MdCYP14 | Chr15 | MdCYP25 |
| Chr13 | MdCYP21 | Chr16 | MdCYP28 |
| Chr13 | MdCYP22 | Chr16 | MdCYP29 |

**Supplementary Table 4**

The intergenomic duplications between

| ***M. domestica*** | ***M. sieversii*** |
| --- | --- |
| MD05G1119400 | Msi_10g012630 |
| MD05G1222400 | Msi_10g020320 |
| MD05G1119400 | Msi_05g013260 |
| MD05G1222400 | Msi_05g021710 |
| MD07G1221700 | Msi_01g013820 |
| MD07G1221700 | Msi_11g006200 |
| MD07G1046700 | Msi_02g023940 |
| MD07G1046700 | Msi_07g003880 |
| MD07G1221700 | Msi_07g018130 |
| MD08G1066000 | Msi_15g005560 |
| MD08G1066000 | Msi_08g005980 |
| MD08G1145000 | Msi_08g013540 |
| MD10G1123000 | Msi_10g012630 |
| MD10G1204000 | Msi_10g020320 |
| MD10G1123000 | Msi_05g013260 |
| MD10G1204000 | Msi_05g021710 |
| MD11G1277600 | Msi_11g022980 |
| MD11G1073900 | Msi_11g006200 |
| MD11G1073900 | Msi_03g006070 |
| MD11G1277600 | Msi_03g022400 |
| MD12G1030900 | Msi_12g003230 |
| MD12G1030900 | Msi_14g004180 |
| MD13G1074200 | Msi_13g006640 |
| MD13G1123100 | Msi_13g010870 |
| MD13G1074200 | Msi_16g007000 |
| MD13G1123100 | Msi_16g011360 |
| MD14G1031400 | Msi_12g003230 |
| MD14G1031400 | Msi_14g004180 |
| MD15G1055400 | Msi_15g005560 |
| MD15G1280000 | Msi_15g025490 |
| MD15G1280000 | Msi_02g014380 |
| MD15G1120600 | Msi_08g013540 |
| MD15G1055400 | Msi_08g005980 |
| MD16G1075600 | Msi_13g006640 |
| MD16G1123800 | Msi_13g010870 |
| MD16G1075600 | Msi_16g007000 |
| MD16G1123800 | Msi_16g011360 |
| MD17G1286000 | Msi_17g024870 |
| ***M. domestica*** | ***M. sylvestris*** |
| MD05G1119400 | Msy_10g012070 |
| MD05G1222400 | Msy_10g019320 |
| MD05G1119400 | Msy_05g011960 |
| MD05G1222400 | Msy_05g020450 |
| MD07G1221700 | Msy_01g014190 |
| MD07G1221700 | Msy_01g021290 |
| MD07G1221700 | Msy_11g006090 |
| MD07G1046700 | Msy_02g024900 |
| MD07G1046700 | Msy_07g003430 |
| MD07G1221700 | Msy_07g019120 |
| MD08G1145000 | Msy_15g010200 |
| MD08G1066000 | Msy_15g004720 |
| MD08G1066000 | Msy_08g005940 |
| MD08G1145000 | Msy_08g013180 |
| MD10G1123000 | Msy_10g012070 |
| MD10G1204000 | Msy_10g019320 |
| MD10G1123000 | Msy_05g011960 |
| MD10G1204000 | Msy_05g020450 |
| MD11G1073900 | Msy_11g006090 |
| MD11G1277600 | Msy_11g024380 |
| MD11G1277600 | Msy_03g023600 |
| MD11G1073900 | Msy_03g006410 |
| MD11G1073900 | Msy_07g019120 |
| MD12G1030900 | Msy_12g003190 |
| MD12G1030900 | Msy_14g003070 |
| MD13G1074200 | Msy_13g005760 |
| MD13G1123100 | Msy_13g009670 |
| MD13G1074200 | Msy_16g005620 |
| MD13G1123100 | Msy_16g009510 |
| MD14G1031400 | Msy_12g003190 |
| MD14G1031400 | Msy_14g003070 |
| MD15G1055400 | Msy_15g004720 |
| MD15G1120600 | Msy_15g010200 |
| MD15G1280000 | Msy_15g023720 |
| MD15G1280000 | Msy_02g015000 |
| MD15G1280000 | Msy_05g008370 |
| MD15G1120600 | Msy_08g013180 |
| MD15G1055400 | Msy_08g005940 |
| MD16G1075600 | Msy_13g005760 |
| MD16G1123800 | Msy_13g009670 |
| MD16G1075600 | Msy_16g005620 |
| MD16G1123800 | Msy_16g009510 |
| MD17G1286000 | Msy_17g024970 |
| ***M. domestica*** | ***M. prunifolia*** |
| MD05G1222400 | MP05G1210500.1 |
| MD05G1222400 | MP10G1191800.1 |
| MD07G1046700 | MP02G1238300.1 |
| MD07G1046700 | MP07G1033900.1 |
| MD08G1066000 | MP08G1060000.1 |
| MD08G1145000 | MP08G1131500.1 |
| MD08G1145000 | MP15G1110000.1 |
| MD08G1066000 | MP15G1054100.1 |
| MD10G1204000 | MP05G1210500.1 |
| MD10G1204000 | MP10G1191800.1 |
| MD11G1277600 | MP11G1236600.1 |
| MD12G1030900 | MP12G1029000.1 |
| MD12G1030900 | MP14G1027100.1 |
| MD13G1123100 | MP13G1108600.1 |
| MD13G1074200 | MP13G1072800.1 |
| MD13G1074200 | MP16G1069300.1 |
| MD13G1123100 | MP16G1113900.1 |
| MD14G1031400 | MP12G1029000.1 |
| MD14G1031400 | MP14G1027100.1 |
| MD15G1120600 | MP08G1131500.1 |
| MD15G1055400 | MP08G1060000.1 |
| MD15G1280000 | MP15G1250500.3 |
| MD15G1120600 | MP15G1110000.1 |
| MD15G1055400 | MP15G1054100.1 |
| MD16G1123800 | MP13G1108600.1 |
| MD16G1075600 | MP13G1072800.1 |
| MD16G1075600 | MP16G1069300.1 |
| MD16G1123800 | MP16G1113900.1 |
| MD17G1286000 | MP17G1278600.1 |

**Supplementary Table 5**

The qRT-PCR primer sequences of *MdCYPs*.

| Gene | Primer |
| --- | --- |
| MdCYP1-F | AGGAAAGCGATTTGGAGCAA |
| MdCYP1-R | GCTCGGAAATTCTCTGTGGT |
| MdCYP2-F | CATACCGCTAACGTTCCACA |
| MdCYP2-R | TTCCTGTTGTCTTCACAGCC |
| MdCYP3-F | GTACACCGTGTTTGGCAAAG |
| MdCYP3-R | AAGCGGGTTAGCATGTATGG |
| MdCYP4-F | GTCAATGGCAAATGCTGGTC |
| MdCYP4-R | CCATTCCAGACAGCACCTTT |
| MdCYP5-F | CCCACCTTGGTCCAATTGAA |
| MdCYP5-R | CTCTGTGCGAATCATGACCA |
| MdCYP6-F | GTCCTGGTGGTTATCGTGTC |
| MdCYP6-R | TTACAGGAGGTCGGTCAACT |
| MdCYP7-F | CAAAACCCCGAAAACTGCTG |
| MdCYP7-R | ATCTCCACCCTGACACATGA |
| MdCYP8-F | TCACAAACAGCTCTGCTTGT |
| MdCYP8-R | CATCAACATCGTTAAGCGCC |
| MdCYP9-F | GGCAAAGGTGTTAAGGGGAA |
| MdCYP9-R | TCTCATCAGGAAATGTGCCG |
| MdCYP10-F | GTGCACTTGATCACATTGCC |
| MdCYP10-R | CACCACCACGCTGTACATTA |
| MdCYP11-F | ATGTTGTGTTCGGGAGAGTG |
| MdCYP11-R | ACACAAGCTAGTTTAGGGCG |
| MdCYP12-F | TCGTATTTGGCAAGGTGCTT |
| MdCYP12-R | ATTGGAAGTTCGCCACTGTT |
| MdCYP13-F | CCGGTACACCCAATTCGAAT |
| MdCYP13-R | ATCCCCAGTTACCTTTCCGA |
| MdCYP14-F | AGAAAACCGCTGCACTACAA |
| MdCYP14-R | GAGCACAGGTGACTCATCTG |
| MdCYP15-F | CGTCCTCGCCATTTTCCTAA |
| MdCYP15-R | TACCTAAGTGCTGTCCGTCA |
| MdCYP16-F | GGTGCACTTGATCACATTGC |
| MdCYP16-R | CACCACGCTGTCCATTATCA |
| MdCYP17-F | ACTGGTTGGATGGCAAACAT |
| MdCYP17-R | ATCACGACTTTGCTCTTGGG |
| MdCYP18-F | CCCACCTTGGTCCAATTGAA |
| MdCYP18-R | CTCTGTGCGAATCATGACCA |
| MdCYP19-F | GGGGGTTTGTCAAAAAGCAC |
| MdCYP19-R | GGAATTACGGAGTGAACGCT |
| MdCYP20-F | CTGGAGCTGGCATCTTATCG |
| MdCYP20-R | CTCCATTCCTGTGCAGATCC |
| MdCYP21-F | CATCGGGAAGGGATTTTCGT |
| MdCYP21-R | CCTCTGCTTCATGACAGACC |
| MdCYP22-F | ATATGCACGGTCAAGACACC |
| MdCYP22-R | CTTTGTAGGGCGATCACCTC |
| MdCYP23-F | CGAATGCTGGTCCGAATACA |
| MdCYP23-R | TATCAGTCTGGACACTGCCA |
| MdCYP24-F | CCGGTACACCCAATTCGAAT |
| MdCYP24-R | AGTTTCGAGTTCACCCAACC |
| MdCYP25-F | GTTGTTCCCAAGACTGCAGA |
| MdCYP25-R | TCGCCACCCTGTGCTATATA |
| MdCYP26-F | GCTAATAAGGGCCCTGACAC |
| MdCYP26-R | TGATCACCTTTCCAAACGCA |
| MdCYP27-F | TACGCCATTTGAAGACCCAG |
| MdCYP27-R | TGTGGAACGTTAGCGGTATG |
| MdCYP28-F | ACCGAGCGCATTACAATTCT |
| MdCYP28-R | GATTTCAATGGCAGCTGCAG |
| MdCYP29-F | TTCAAGGAGGGGACTTCGAT |
| MdCYP29-R | GCTAACAACTCCTGGTCCAG |
| MdCYP30-F | CTCTTTCCACCGCATCATCA |
| MdCYP30-R | GGACCAACATGCTTCAATGC |

**Supplementary Table 6**

Primer of coding sequence and promoter sequence of *MdCYP16*.

| MdCYP16-F | ATGTCAGTGCTTATAGTGAC |
| --- | --- |
| MdCYP16-R | TCGCCTTCTATGTTCTTTTT |
| pENTR-MdCYP16-F | AAGGAGCCCTTCACCATGTCAGTGCTTATAGTGAC |
| pENTR-MdCYP16-R | GGCGCGCCCCCTTTCGCCTTCTATGTTCTTTTT |
| ProMdCYP16-F | ACCATTCATTGCCGAGGATA |
| ProMdCYP16-R | GGTCTCTCACTGCATTCAAC |
| ProMdCYP16::GUS-F | CCGGAATTCCCGGGGACCATTCATTGCCGAGGATA |
| ProMdCYP16::GUS-R | TAGCTTGGCTGCAGGGGTCTCTCACTGCATTCAAC |
